# Relationship between Metabolic Fluxes and Sequence-Derived Properties of Enzymes

**DOI:** 10.1155/2014/817102

**Published:** 2014-10-29

**Authors:** Peteris Zikmanis, Inara Kampenusa

**Affiliations:** Institute of Microbiology and Biotechnology, University of Latvia, Kronvalda Boulevard 4, Riga LV-1010, Latvia

## Abstract

Metabolic fluxes are key parameters of metabolic pathways being closely related to the kinetic properties of enzymes, thereby could be dependent on. This study examines possible relationships between the metabolic fluxes and the physical-chemical/structural features of enzymes from the yeast *Saccharomyces cerevisiae* glycolysis pathway. Metabolic fluxes were quantified by the COPASI tool using the kinetic models of Hynne and Teusink at varied concentrations of external glucose. The enzyme sequences were taken from the UniProtKB and the average amino acid (AA) properties were computed using the set of Georgiev's uncorrelated scales that satisfy the VARIMAX criterion and specific AA indices that show the highest correlations with those. Multiple linear regressions (88.41% <*R*_adjusted_^2^ < 93.32%; *P* < 0.00001) were found between the values of metabolic fluxes and the selected sets of the average AA properties. The hydrophobicity, *α*-helicity, and net charge were pointed out as the most influential characteristics of the sequences. The results provide an evidence that metabolic fluxes of the yeast glycolysis pathway are closely related to certain physical-chemical properties of relevant enzymes and support the view on the interdependence of catalytic, binding, and structural AA residues to ensure the efficiency of biocatalysts and, hence, physiologically adequate metabolic processes.

## 1. Introduction

The general concepts and methods of systems biology become increasingly important in modern microbiology research. In terms of “systems microbiology,” this approach allows to analyze and describe as a whole the molecular interactions that occur within a microbial cell or community [1–3]. The complex physiological expressions of microorganisms, in turn, can best be described by the levels and distribution of metabolic fluxes. They are considered as the key parameters of any metabolic pathway and, hence, the fundamental determinants of cell physiology [2, 4]. On the other hand, enzyme activity is one of the major factors influencing the magnitude of metabolic fluxes in any cell [5]. According to concepts of systems biology, metabolic fluxes are net sums of underlying enzymatic reaction rates represented by integral outputs of three biological quantities which interact at the level of enzyme kinetics: kinetics parameters as well as enzyme and reactant concentrations [1]. Furthermore, an integrated view on enzymes suggests considering them as dynamic assemblies whose variable structures are closely related to catalytic functions [6]. It is, therefore, an important task to extend the knowledge on the enzyme sequence, structure, and function relationship for understanding the physiological dynamics within the cell. In particular, it is necessary to create comprehensive, quantitative and predictive models that enhance an understanding of cellular behaviour under varied environment in compliance with the central aim of systems biology. These goals, in turn, need certain specifications to define variables for potent quantitative relationships. According to the above notions, the quantified levels of metabolic fluxes as well as the kinetic parameters of enzymes should be considered as appropriate functional characteristics of protein sequences [1] and, hence, as a proper response, that is, dependent, variables for such relationships. The specification of explanatory, that is, independent, variables in terms of sequence-dependent properties are much more complicated, since they could involve a lot of equally possible indices. Nevertheless, there are solid grounds to distinguish the amino acid (AA) composition (AAC) of proteins among a variety of related quantitative characteristics of the sequences. AAC is a simplest attribute of proteins among all potential sequence descriptors which represents the frequencies of occurrence of the natural AA thereby creating a 20-dimensional feature space for a given protein sequence [7]. Nevertheless, it appears as a simple, yet powerful feature for a successful prediction of versatile protein properties, including protein folding and mutual interactions [8–10]. This allowed the hypothesis that it is possible to push further the use of AAC to describe protein sequences relative to their functions. Our previous studies confirm this possibility, since the relationship between the kinetic constants of the yeast* Saccharomyces cerevisiae* glycolytic enzymes and the AAC of corresponding sequences has been found [11], as well as statistically robust multivariate regression models established which link both the flux distribution through the glycolysis pathway and the AAC of respective enzymes [12]. At the same time, it should be noted that present results reflect only the first level, looking at a much wider set of potential relationships, as they do not give enough insights into the structural and physical-chemical properties of sequences as well as the actual contribution of AA [13].

However, the possible solutions have been defined as rather difficult task, arising from the sequence metrics problems [14, 15]. In fact, to apply multivariate methods of analysis it is necessary to convert the AA sequences of proteins into metric terms which represent structural features and/or physical-chemical properties remaining outside the view if AAC is used as a simple metric [13]. Such an expression of sequences is known as the property vectors [7, 15] which may include a set of numerical descriptors for each individual AA or the average AA property for each protein [16] to describe full sequences. The propensity scores for diverse AA properties (physical-chemical, conformational, energetic, etc.) are summarized in the AAindex database (currently 544 indices) [17] and widely used as appropriate numerical descriptors. On the other hand, rigorous numerical analysis of the protein characteristics requires the property vectors that are both complete and nonredundant, which is hardly accessible using arbitrarily chosen sets of properties due to the abundance of interrelated data in the AAindex database [7, 15]. To overcome such a situation, suitable approaches based on the factor analysis of the AA property scores have been developed. Thus, several groups of mutually related properties as the orthogonal “factors” have been defined and then attributed to more general numerical scores in respect of individual AA [7, 15, 18, 19]. As a result, a sharp reduction of dimensionality can be achieved thus gaining the potential use to get the Quantitative Sequence-Activity Models (QSAM) of proteins. In general, this approach aims to predict the outcome of the response variables from a set of adequately chosen explanatory variables using the appropriate regression models in terms of mathematical equations [14].

Therefore, following our previous line of research, the goal of the present study is to test the possible relationship (QSAM) between the flux distribution through the yeast* Saccharomyces cerevisiae* glycolysis pathway and the physical-chemical/structural features of enzyme sequences.

## 2. Material and Methods

### 2.1. Dataset Formation

The data set consisted of the amino acid (AA) sequences, representing the enzymes/carriers for the core reactions of the yeast* Saccharomyces cerevisiae* glycolysis pathway: low-affinity glucose transporter (HXT1, P32465), hexokinase (HXK, EC 2.7.1.1, P04806), glucose-6-phosphate isomerase (GPI, EC 5.3.1.9, P12709), 6-phosphofructo-2-kinase (PFK1, EC 2.7.1.105, P40433), fructose-biphosphate aldolase (ALD1, EC 4.1.2.13, P14540), triose-phosphate isomerase (TIM, EC 5.3.1.1, P00942), glyceraldehyde-3-phosphate dehydrogenase (GAPDH1, EC 1.2.1.12, P00360), 3-phosphoglycerate kinase (PGK1, EC 2.7.2.3, P00560), phosphoglycerate mutase (PGM, EC 5.4.2.1, P00950), enolase (ENOL, EC 4.2.1.11, P00924), pyruvate kinase (PK1, EC 2.7.1.40, P00549), pyruvate decarboxylase (isozyme1, PDC, EC 4.1.1.1, P06169), and alcohol dehydrogenase (ADH1, EC 1.1.1.1, P00330), as well as the main branches that include glycogen synthase (isoform1, EC 2.4.1.11, P23337) and glycerol-3- phosphate dehydrogenase (GPD1, EC 1.1.1.8, Q00055) together with enzymes involved into the turnover of ATP: plasma membrane ATPase 1 (EC 3.6.3.6, P05030), adenylate kinase 1 (AK1, EC 2.7.4.3, P07170).

The protein AA sequences were taken from the UniProtKB (http://www.uniprot.org) database under accession numbers as indicated in the brackets above. The AA composition (frequencies of AA occurrence) of sequences (AAC) was computed using ExPASy/ProtParam (http://web.expasy.org/protparam/) tool.

Metabolic fluxes within the yeast* Saccharomyces cerevisiae *glycolysis pathway were estimated using the kinetic models of Hynne [20] and Teusink [21] from the BioModels Database (http://www.ebi.ac.uk/biomodels/)—BIOMD0000000061 and BIOMD0000000064, respectively. Simulation experiments were performed for both models using the COmplex PAthway Simulator tool (Copasi 4.7 Build 34, http://www.copasi.org) at perturbed initial concentrations of external glucose (25 mM, 50 mM, and 100 mM).

The enzyme AAC was converted into a feature-based numerical representation using the average AA property for each sequence and computed according to the standard formula [16] (see (1):

(1) $$\begin{array}{c} P_{\text{ave}}\left(i\right)=\overset{N}{\underset{j=1}{\sum }}\frac{P\left(j\right)}{N}, \end{array}$$

where *P*_ave_(*i*) is the average AA property for each sequence and *P*(*j*) is the property value for *j*th residue and the summation over *N*, the total number of residues in a protein. The transformation of sequences into fixed-size numerical feature vectors was performed in two steps. First, the average AA property for each sequence was estimated using the generalized numerical scores in respect of individual AA [18]. Appropriate orthogonal scales, based on the factor analysis for ten groups of mutually related AA properties from the AAindex database and satisfying the VARIMAX criterion, have been proposed as the interpretable numerical descriptors of the protein AA space [18]. This led to a set of 10D numerical vectors that represents generalized physical-chemical and structural features of sequences. The relevant data are summarized in the Supplementary Information 1: Table  S1-1 and Table S1-2 available online at http://dx.doi.org/10.1155/2014/817102. Second, the average AA property for each sequence was computed using the narrower sets of specific AA indices from generalized AA property groups with a highest correlation to the VARIMAX-derived scales [18]. The relevant data are summarized in the Supplementary Information 1: Table S1-3, Table S1-4. A choice of specific AA indices, representing the generalized AA property groups, was based on the findings of multivariate analysis for the previously obtained set of 10D vectors carried out as described below.

The accession numbers (AAindex database), description, and designation of specific AA indices which were used for this purpose are represented in Table 1.

### 2.2. Data Processing and Multivariate Analysis

The data representing metabolic fluxes, as the dependent variables and the sets of numerical property vectors for respective enzymes, as the independent variables (Table S1-1, Table S1-2, Table S1-3, and Table S1-4) were processed by correlation analysis (parametric and nonparametric) using the Statgraphics Plus (Manugistics Inc., Mar., USA) and SPSS 11.0 for Windows (SPSS Inc., Ill., USA) and subjected to the multiple linear regression analysis using the same software. Explanatory variables in the models were subsequently checked by stepwise forward selection procedures thus finding the significant one-variable models as well as significant two-variable models to arrange all the variables in groups of 2 at a time for each model. The best three-variable models were formed by adding another variable one by one from the remaining variables, and the variables that yielded the greatest increase in the adjusted *R*-square value besides keeping the variance inflation factor (VIF) below the threshold value of 3.3 [22] or, in exceptional cases, 5.0, were included. If the VIF values drew near or exceeded these limits the Ridge regression (Statgraphics Plus) with the varied parameter values was employed to check the actual adjusted *R*-square criterion of regression models. This process was repeated to obtain four-variable and larger models until no variables could increase the adjusted *R*-square value.

In addition, the corrected Akaike's (AICc) information criterion [23] was used to verify that the appropriate explanatory variables have been selected. Fisher's *F*-test for analysis of variance (ANOVA) was employed to evaluate the statistical significance of regression models and Student's *t*-test was used to check the significance of regression coefficients. The leave-one-out cross-validation (LOOCV) procedure was used to validate developed regression models [24]. The linear plots of the metabolic fluxes estimated by kinetic models against those predicted by the multiple regression models were used throughout the study to assess the fit for observed multivariate relationships according to adjusted *R*-square values.

The *P* values < 0.05 were considered to be statistically significant for parametric and nonparametric tests.

## 3. Results

**Figure 1:**
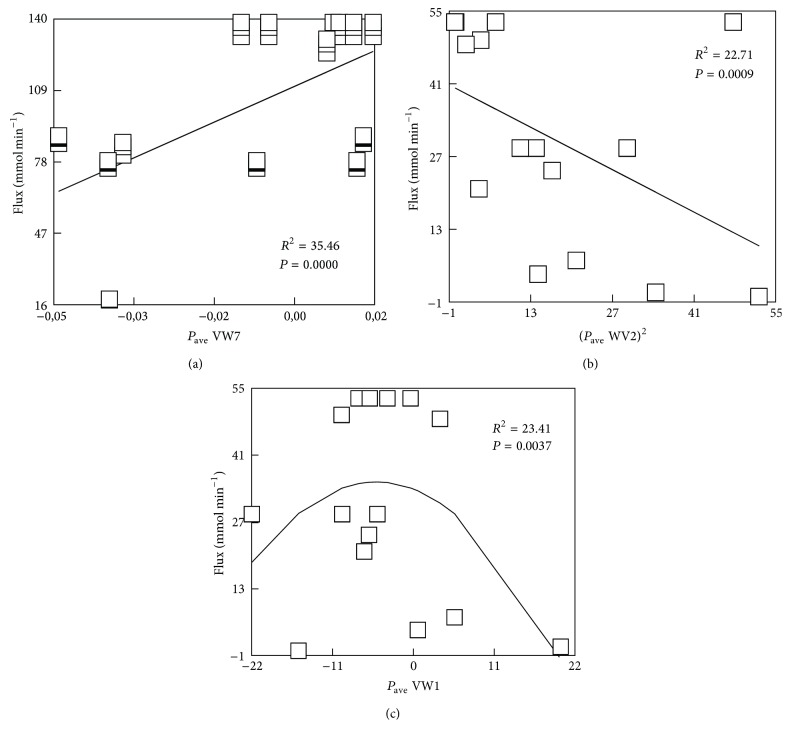
Linear and nonlinear pair correlations between the metabolic fluxes and the average AA properties of the yeast* Saccharomyces cerevisiae* enzyme sequences, as specified in Table 1; the data represent Teusink's (a) and Hynne's ((b), (c)) models I and II, respectively (Table 2). The correlations are significant at the nonparametric assessment (Kendall's *τ*, Spearman's *ρ* correlation coefficients).

**Figure 2:**
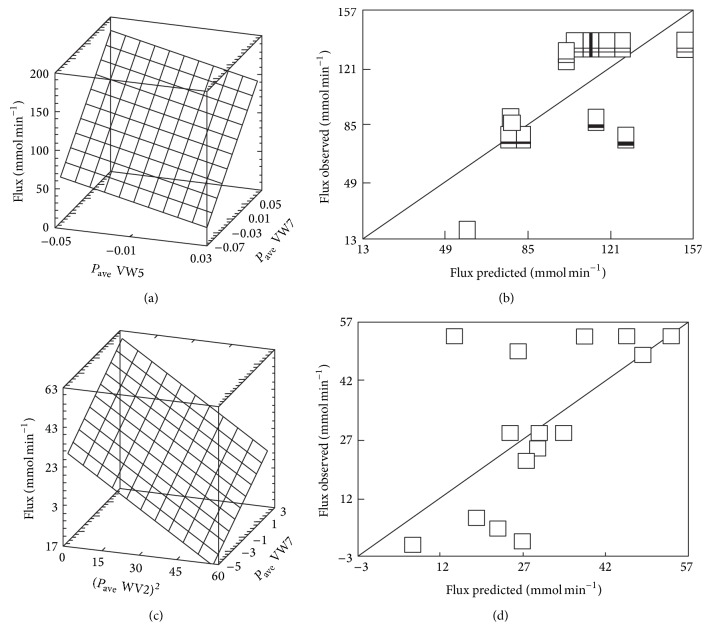
The multiple linear regressions showing changes of the metabolic fluxes as dependent variables upon the two average AA properties of the yeast* Saccharomyces cerevisiae* enzyme sequences, as specified in the Table 1. The data ((a), (c)) represent models I and II, respectively (Table 2). The observed versus predicted plots ((b), (d)) for the values of dependent variables ((a) and (c), resp.). The predicted values were calculated from the regression equations: flux (model I) = Flux: 108.975 + 988.917∗*P*_ave_WV7 − 553.390∗*P*_ave_WV5 (*R*_adj._^2^ = 47.84%, *P* = 0.0000); flux (model II) = 47.576 − 0.757∗(*P*_ave_WV2)^2^ + 3.696∗*P*_ave_WV7 (*R*_adj._^2^ = 35.71%, *P* = 0.0000). All the multiple and pair correlations ((a), (b), (c), (d)) are significant at the nonparametric assessment (Kendall's *τ*, Spearman's *ρ* correlation coefficients).

Statistical analysis of the data set (Table S1-1, Table S1-2) revealed a number of significant pair correlations (Figure 1) between the levels of metabolic fluxes through the yeast* Saccharomyces cerevisiae* glycolysis pathway and the values of individual average amino acid (AA) properties for respective enzymes expressed according to VARIMAX scales [18] and, therefore, representing the groups (Table 1) of generalized AA features. In addition, the correlations also appeared using the squares of these properties (Figures 1 and 2). Therefore, for further analysis and parameter selection, the data set also included the squared values of the average AA properties.

**Figure 3:**
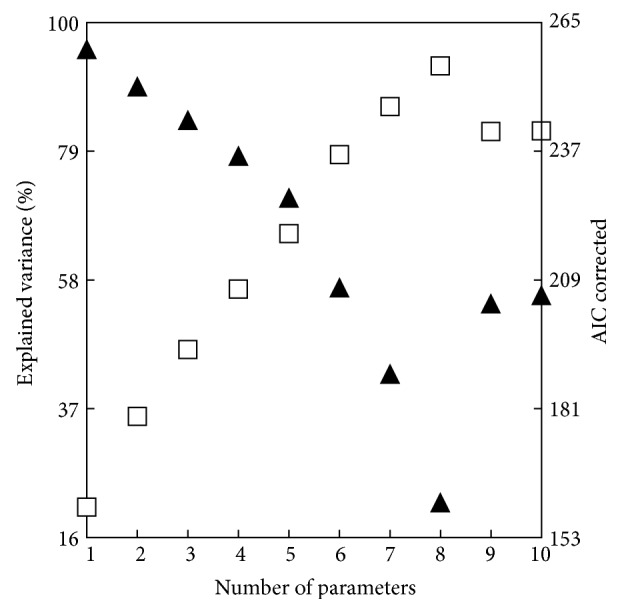
The changes in the percentage of explained variance (□) and the values of corrected Akaike's information criterion (AIC c) (▲) on the growing number of independent variables (the average AA properties of enzyme sequences) included in the multiple regression. Variables in the model (Tables 1 and 2, model II): 1—(*P*_ave_WV2)^2^, 2—(*P*_ave_WV2)^2^, *P*_ave_WV7, 3—(*P*_ave_WV2)^2^, *P*_ave_WV7, *P*_ave_WV1, 4—(*P*_ave_WV2)^2^, *P*_ave_WV7, *P*_ave_WV1, (*P*_ave_WV1)^2^, 5—(*P*_ave_WV2)^2^, *P*_ave_WV7, *P*_ave_WV1, (*P*_ave_WV1)^2^, (*P*_ave_WV5)^2^, 6—(*P*_ave_WV2)^2^, *P*_ave_WV7, *P*_ave_WV1, (*P*_ave_WV1)^2^, (*P*_ave_WV5)^2^, *P*_ave_WV5, 7—(*P*_ave_WV2)^2^, *P*_ave_WV7, *P*_ave_WV1, (*P*_ave_WV1)^2^, (*P*_ave_WV5)^2^, *P*_ave_WV5, *P*_ave_WV6, 8—(*P*_ave_WV2)^2^, *P*_ave_WV7, *P*_ave_WV1, (*P*_ave_WV1)^2^, (*P*_ave_WV5)^2^, *P*_ave_WV5, *P*_ave_WV6, (*P*_ave_WV3)^2^, 9—(*P*_ave_WV2)^2^, *P*_ave_WV7, *P*_ave_WV1, (*P*_ave_WV1)^2^, (*P*_ave_WV5)^2^, *P*_ave_WV5, *P*_ave_WV6, (*P*_ave_WV3)^2^, *P*_ave_WV10^a^, 10—(*P*_ave_WV2)^2^, *P*_ave_WV7, *P*_ave_WV1, (*P*_ave_WV1)^2^, (*P*_ave_WV5)^2^, *P*_ave_WV5, *P*_ave_WV6, (*P*_ave_WV3)^2^, *P*_ave_WV10, *P*_ave_WV9^b^. (a) The scale WV10 correlates with the NMR parameters and pK values of AA [18]. (b) The scale WV9 correlates with the indices of protein backbone topography and relative mutability of AA [18].

Subsequent analysis of the data showed that the stepwise inclusion of additional variables leads to a statistically significant multiple regression, where the metabolic fluxes depend on two or more average AA properties of the respective enzymes, thus substantially increasing the proportion of the “explained” variance (Figures 2 and 3). The increasing adjusted *R*-square values (Figure 3) indicate that the “explained” variance substantially rises with the growing number of variables in the regression model, although in a somewhat nonlinear proportion due to a more pronounced contribution of the few most powerful AA properties (Figure 3), which is also well reflected in the relevant changes of the corrected Akaike's information criterion (AIC c). Such an uneven impact of variables also follows from the different values of the standardized regression coefficients (Tables 2 and 3). If additional variables were included in the models, the results did not improve, due to sharply growing values of variance inflation factor (VIF). Thus, in order to keep them below the desirable threshold of 3.3 [22], it was necessary to use the Ridge regression for an adequate modelling.

However, the appropriate Ridge parameter values (*λ* = 0.02 to 0.04) caused a marked decrease in actual *R*-square levels, as well as an increase of AIC c, which indicates a certain decline in the quality of extended models (Figure 3).

Thus, in the present case seven (model I) or eight (model II) variables proved to be adequate to provide statistically robust multiple linear regression models linking the values of metabolic fluxes predicted by different kinetic models [20, 21] with the average AA characteristics of corresponding sequences (Table 2), expressed on the grouped [18] physical-chemical or structural properties. It should be noted that the total number of variables exceeds the actual number of effective sequence-derived properties since regression models include both linear and quadratic terms of them. Both models include nearly the same variables, although with differing effects, where the groups 7, 2, and 5 (Figures 1 and 2, Tables 1 and 2) appeared as most influential for the average AA properties of enzyme sequences. These groups, in terms of proposed VARIMAX scales, correlate well with the AA natural indices such as the isoelectric point (group 7), the *α*-helicity (group 2), and the measure of linker propensity (group 5) [18].

Further step of the study was carried out in a similar way. The average AA property for each enzyme sequence was computed using the specific AA indices [17] from generalized AA property groups (Table 1) with a highest correlation to the VARIMAX-derived scales [18]. Subsequent analysis of the data (Table S1-3, Table S1-4) including pair and multiple correlations and stepwise parameter selection as well as monitoring the steps by the VIF and adjusted *R*-square values of corresponding Ridge regression, as described above, led to statistically robust multiple linear regressions (Table 3).

The resulting models, therefore, link the metabolic fluxes of the yeast* Saccharomyces cerevisiae* glycolysis pathway to defined characteristics of respective enzymes in terms of the average AA property [16] for each protein. As in the case of generalized property groups (Tables 1 and 2), these models (Table 3) represent similar sets of independent variables, although with a different layout for their impact. Thus, if the metabolic fluxes were determined (model III) by Teusink's kinetic model (Table 3), the net charge and the normalized relative frequency of *α*-helix (Table 1) turned out to be the most important properties of enzyme sequences. In other case, the use of Hynne's model revealed (model IV) two hydrophobicity-based properties together with the normalized frequency of *α*-helix (Table 1) from CF (a set of 33 proteins) as the most influential features of enzymes (Table 3).

The matching quality of the data obtained by the proposed models was evaluated by the linear plots (Supplementary Information 2: Figure  S2) of the metabolic fluxes estimated by kinetic models against those predicted by regression models (Tables 2 and 3). The highly significant adjusted *R*-square values also indicate that the models (Tables 2 and 3) adequately represent the actual relationships between the sequence-derived properties of enzymes and the values of metabolic fluxes, since only a relatively small proportion (6.68–11.59%) of the total variance remains unexplained. The results of variance analysis (ANOVA) together with the confidence intervals for the regression models are summarized in the Supplementary Information 2: Table S2-1 and Table S2-2, respectively.

The validation of models using the leave-one-out cross-validation procedure (LOOCV) [24] showed only a slight effect on the *R*-square values (Tables 2 and 3, Figure S2), which still remain highly significant (*P* < 0.00001). Besides, the observed values of the variance inflation factor (VIF) (Tables 2 and 3) indicate that a relatively small collinearity of independent variables can not substantially affect the observed multivariate relationships [22]. It should be noted that similar statistically robust relationships can be established also for the data listed in other sources. In particular, using the values of metabolic fluxes, which fit in well with both the recently developed [25] standard model built without regulatory information and the model with an integrated regulatory information regarding the yeast* Saccharomyces cerevisiae* metabolic network. These results are summarized in Supplementary Information 3: Table S3 and Figure S3.

## 4. Discussion 

The obtained results indicate that the metabolic fluxes determined by Hynne's and Teusink's full-scale models for the yeast* Saccharomyces cerevisiae* glycolysis pathway appear as closely related to the sequence-derived properties of implicated enzymes.

The relevant multivariate regression models (Tables 2 and 3) show that a representation of enzymes as the numerical vectors, which include, in accordance with the interpretable multiscale descriptors [18], the average AA properties of each protein [16], is appropriate to promote the use of computational methods for turning protein sequence data into the functional knowledge that is an important task to understand complex biological systems [26].

On the other hand, such an approach meets the general lines for the multiscale nature and, consequently, the scale-space representation of real-world objects [27], which implies that any of them may be perceived in different ways depending on the scale of observation that is also fully attributable to complex biological sequences.

Different sets of the predictor variables in the regression models (Tables 2 and 3), as well as the varied flux distributions of both kinetic models used in this study likely reflect the fact that the models have been developed to describe the glycolysis under diverse experimental conditions and even for different yeast strains; therefore, the approaches of model building also differed [28].

This study in the most direct way continues our previous research [11, 12] thereby forming the mutually affirmative and complementary set of results. In addition, the resulting relationships are well in line with the views of the actual interdependence of catalytic, binding, and structural residues to ensure the full-scale efficiency of biocatalysts [29] supported by the findings that a certain functional overlap may occur between the sets of AA [30] as well as by the evidence confirming that the primary structure-derived features [31, 32] or integral physical-chemical indices of proteins [33] can be used to predict the values of kinetic constants for particular enzymes.

The results thus provide further evidence that the enzyme operation and hence the metabolic fluxes are directly dependent on the general physical-chemical and structural properties of the full enzyme sequences. Therefore, it might be useful for structure/function studies to look more beyond the active centre composition of the enzyme but carefully assess the overall physical and chemical properties of biocatalysts. The more so that the modest success of creating artificial enzymes also points to currently unknown, probably crucial, parameters that could significantly affect enzyme catalysis [34]. In particular, the results give some grounds to believe that it is possible to reduce the size of artificial enzymes if the overall AA composition and, hence, their physical-chemical properties remain very similar to the baseline enzymes.

It is clearly understood that the estimates of metabolic fluxes currently obtained by the kinetic models represent an approximation, albeit realistic enough, of their “true” values which could involve multiple regulatory mechanisms including gene expression and posttranslational modification [1, 25, 35]. On the other hand, such an approximation can also facilitate the search for sequence and activity relationships of respective enzymes, since in that case the masking effect of such overlapping factors can be “removed.” In other words, it provides an opportunity to outline a desirable, even necessary, but certainly not sufficient yet precondition for the efficient distribution of actual metabolic fluxes according to the physical-chemical and structural properties of enzymes. In this respect, the recently launched attempts to progressively incorporate such regulatory information into the yeast* Saccharomyces cerevisiae* kinetic model [25] can provide a basis for further research in the field.

## 5. Conclusion

The obtained sequence-activity relationships in the form of quantitative models (QSAM) will allow to assess the contribution of individual AA properties to the overall compositional features of proteins and thus to specify potential targets within enzyme sequences in order to attain a purposeful modification of biocatalysts and, hence, the metabolic fluxes in microorganisms, particularly if there is a need to include or replace (e.g., metabolic engineering, dynamic modelling) any additional enzyme currently not represented in a given metabolic pathway, which is essential for metabolic engineering and synthetic biology.

## Supplementary Material

Supplementary information 1:
Table S1-1: The dataset used in multivariate analysis which comprises metabolic fluxes and numerical vectors representing the average amino acid (AA) property for each enzyme sequence (Teusink's kinetic model, VARIMAX scales).
Table S1-2: The dataset used in multivariate analysis which comprises metabolic fluxes and numerical vectors representing the average AA property for each enzyme sequence (Hynne's kinetic model, VARIMAX scales).
Table S1-3: The dataset used in multivariate analysis which comprises metabolic fluxes and numerical vectors representing the average AA property for each enzyme sequence (Teusink's kinetic model, specific AA indices).
Table S1-4: The dataset used in multivariate analysis which comprises metabolic fluxes and numerical vectors representing the average AA property for each enzyme sequence (Hynne's kinetic model, specific AA indices).
Supplementary information 2:
Figure S2: The linear plots for the metabolic fluxes of the yeast Saccharomyces cerevisiae glycolysis pathway estimated by kinetic models against those predicted by linear regression models I-IV and by the leave-one-out cross-validation (LOOCV) of the models.
Table S2-1: The variance analysis of the regression models.
Table S2-2: Standard errors and confidence intervals for the linear regression models.
Supplementary information 3:
Table S3: Elements and the statistical indices for multiple linear regression model which links the values of metabolic fluxes and the average AA properties of the yeast Saccharomyces cerevisiae enzyme sequences expressed according to the specific AA indices.
Figure S3: The linear plot for the metabolic fluxes of the yeast Saccharomyces cerevisiae glycolysis pathway estimated by kinetic models against those predicted by linear regression model.

## Figures and Tables

**Table 1:** VARIMAX scales and specific AA indices used for the estimation of the average AA property for the yeast *Saccharomyces cerevisiae* enzyme sequence.

AA property group (VARIMAX scale^a^)	Designation for models	Accession number AAindex database	Description
1 (VW1)	1-1	NADH010102	Hydropathy scale based on self-information values in the two-state model (9% accessibility)
	1-2	BIOV880101	Information value for accessibility; average fraction 35%
	1-3	ROSG850102	Mean fractional area loss

2 (VW2)	2-1	PALJ810102	Normalized frequency of *α*-helix from CF (33 proteins)
	2-2	KANM800101	Average relative probability of helix
	2-3	ISOY800101	Normalized relative frequency of *α*-helix

3 (VW3)	3-1	PONJ960101	Average volumes of residues
	3-2	TSAJ990102	Volumes not including the crystallographic waters using the ProtOr
	3-3	FAUJ880103	Normalized van der Waals volume

5 (VW5)	5-1	BUNA790101	*α*-NH chemical shifts
	5-2	FINA910102	Helix initiation parameter at position i, i*þ*1, i*þ*2
	5-3	AURR980119	Normalized positional residue frequency at helix termini *C* ^*ff*^

6 (VW6)	6-1	AURR980117	Normalized positional residue frequency at helix termini *C* ^*f*^
	6-2	FAUJ880107	N.m.r. chemical shift of *α*-carbon
	6-3	RACS820106	Average relative fractional occurrence in ER(*i*)

7 (VW7)	7-1	KLEP840101	Net charge
	7-2	ZIMJ680104	Isoelectric point
	7-3	FINA910103	Helix termination parameter at position j-2, j-1, j

^a^Georgiev, 2009 [18].

**Table 2:** Elements and the statistical indices for multiple linear regression models which link the values of metabolic fluxes and the average AA property for the yeast *Saccharomyces cerevisiae* enzyme sequences, expressed according to the VARIMAX scales.

Model	Dependent^a^ variable	Parameters^b^	Regression coefficient (standardized value)	S.E.	*t* value	*P* value	*R* ^2^, % *R*_adj._^2^, %	VIF^c^
I	Metabolic flux (Teusink's model)	*Constant *	*142.527 *	*3.150 *	*45.25 *	0.0000	94.46	
							93.32	
		*P*_ave_WV7	1749.250 (1.141)	80.843	21.64	0.0000		1.71
		*P*_ave_WV5	−1347.310 (−0.948)	86.853	−15.51	0.0000		2.29
		(*P*_ave_WV1)^2^	−1234.03 (−0.540)	124.491	−9.91	0.0000		1.82
		(*P*_ave_WV5)^2^	−11296.70 (−0.310)	1930.380	−5.85	0.0000		1.73
		(*P*_ave_WV2)^2^	−6350.430 (−0.289)	1137.530	−5.58	0.0000		1.64
		*P*_ave_WV6	202.670 (0.222)	48.639	4.17	0.0002		1.74
		*P*_ave_WV1	44.481 (0.125)	17.534	2.54	0.0159		1.50

II	Metabolic flux (Hynne's model)	*Constant *	*70.629 *	*3.394 *	*20.81 *	0.0000	94.20	
							92.91	
		(*P*_ave_WV2)^2^	−0.780 (−0.638)	0.081	−9.65	0.0000		2.71
		*P*_ave_WV7	8.965 (1.028)	0.575	15.60	0.0000		2.70
		*P*_ave_WV1	−0.836 (−0.396)	0.112	−7.47	0.0000		1.74
		(*P*_ave_WV1)^2^	−0.083 (−0.610)	0.008	−10.11	0.0000		2.26
		(*P*_ave_WV5)^2^	−0.787 (−0.618)	0.076	−10.31	0.0000		2.23
		*P*_ave_WV5	−3.874 (−0.581)	0.383	−10.11	0.0000		2.05
		*P*_ave_WV6	2.683 (0.505)	0.311	8.63	0.0000		2.12
		(*P*_ave_WV3)^2^	−0.085 (−0.414)	0.014	−5.97	0.0000		2.99

^a^Represent the mean values of metabolic fluxes within the range of external glucose concentrations as specified in the “Material and Methods”.

^b^Elements of multiple linear regression which represent the average AA property, as specified in the Table 1, of the yeast *Saccharomyces  cerevisiae* enzyme sequences and the constant (intercept) of equation.

^c^The variance inflation factor which indicates the impact of collinearity between the independent variables [22].

**Table 3:** Elements and the statistical indices for multiple linear regression models which link the values of metabolic fluxes and the average AA properties of the yeast *Saccharomyces cerevisiae* enzyme sequences expressed according to the AAindex scales.

Model	Dependent^a^ variable	Parameters^b^	Regression coefficient (standardized value)	S.E.	*t* value	*P* value	*R* ^2^, % *R*_adj._^2^, %	VIF^c^
III	Metabolic flux (Teusink's model)	*Constant *	*1206.180 *	*188.077 *	*6.41 *	0.0000	94.02	
							93.19	
		(*P*_ave_WV7-1)^2^	−61514.800 (−0.865)	3379.980	−18.20	0.0000		1.36
		*P*_ave_WV2-3	679.041 (0.400)	78.424	8.66	0.0000		1.29
		(*P*_ave_WV5-1)^2^	−17.034 (−0.621)	1.696	−10.05	0.0000		2.30
		(*P*_ave_WV1-1)^2^	−0.070 (−0.378)	0.014	−5.12	0.0000		3.28
		*P*_ave_WV3-1	−4.820 (−0.336)	0.847	−5.69	0.0000		2.10

IV	Metabolic flux (Hynne's model)	*Constant *	*2450.460 *	*187.878 *	*13.04 *	0.0000	90.25	
							88.41	
		(*P*_ave_WV1-1)^2^	−0.181 (−1.570)	0.013	−13.88	0.0000		4.86
		*P*_ave_WV1-3	−2592.690 (−1.202)	182.567	−14.20	0.0000		2.72
		*P*_ave_WV2-1	−466.789 (−0.358)	83.438	−5.60	0.0000		1.55
		(*P*_ave_WV5-1)^2^	−10.664 (−0.610)	1.388	−7.68	0.0000		2.39
		(*P*_ave_WV7-1)^2^	−17453.200 (−0.423)	2559.790	−6.82	0.0000		1.46
		*P*_ave_WV6-1	276.424 (0.360)	49.933	5.54	0.0000		1.61
		*P*_ave_WV3-1	2.648 (0.349)	0.594	4.45	0.0001		2.33

^a^Represent the mean values of metabolic fluxes within the range of external glucose concentrations as specified in the “Material and Methods.”

^b^Elements of multiple linear regression which represent the average AA property, as specified in Table 1, of the yeast *Saccharomyces  cerevisiae* enzyme sequences and the constant (intercept) of equation.

^c^The variance inflation factor which indicates the impact of collinearity between the independent variables [22].
