# Supplementary material for: Relationship between Metabolic Fluxes and Sequence-Derived Properties of Enzymes
Source: Int Sch Res Notices. 2014 Oct 29;2014:817102. doi: 10.1155/2014/817102 (PMC4897147; doi:10.1155/2014/817102)
Supplement: Supplementary file 1 — Supplementary information 1: Table S1-1: The dataset used in multivariate analysis which comprises metabolic fluxes and numerical vectors representing the average amino acid (AA) property for each enzyme sequence (Teusink's kinetic model, VARIMAX scales). Table S1-2: The dataset used in multivariate analysis which comprises metabolic fluxes and numerical vectors representing the average AA property for each enzyme sequence (Hynne's kinetic model, VARIMAX scales). Table S1-3: The dataset used in multivariate analysis which comprises metabolic fluxes and numerical vectors representing the average AA property for each enzyme sequence (Teusink's kinetic model, specific AA indices). Table S1-4: The dataset used in multivariate analysis which comprises metabolic fluxes and numerical vectors representing the average AA property for each enzyme sequence (Hynne's kinetic model, specific AA indices). Supplementary information 2: Figure S2: The linear plots for the metabolic fluxes of the yeast Saccharomyces cerevisiae glycolysis pathway estimated by kinetic models against those predicted by linear regression models I-IV and by the leave-one-out cross-validation (LOOCV) of the models. Table S2-1: The variance analysis of the regression models. Table S2-2: Standard errors and confidence intervals for the linear regression models. Supplementary information 3: Table S3: Elements and the statistical indices for multiple linear regression model which links the values of metabolic fluxes and the average AA properties of the yeast Saccharomyces cerevisiae enzyme sequences expressed according to the specific AA indices. Figure S3: The linear plot for the metabolic fluxes of the yeast Saccharomyces cerevisiae glycolysis pathway estimated by kinetic models against those predicted by linear regression model. [file 817102.f1.zip › manuscript_SI1.pdf]

## RELATIONSHIP BETWEEN METABOLIC FLUXES AND SEQUENCE-DERIVED PROPERTIES OF ENZYMES

**Peteris Zikmanis, Inara Kampenusa**
*Institute of Microbiology and Biotechnology, University of Latvia, Kronvalda Boulevard 4, LV-1010, Riga, Latvia;*
*Correspondence should be addressed to Peteris Zikmanis; peteris.zikmanis@lu.lv*

# Supplementary information 1

**Table S1-1**. The dataset used in multivariate analysis which comprises metabolic fluxes [a] and numerical vectors representing the average amino acid property [b] for each enzyme sequence.

| External glucose, mM | metabolic flux, mmol min $^{-1}$ | $P_{ave}$ WV1 | $P_{ave}$ WV2 | $P_{ave}$ WV3 | $P_{ave}$ WV4 | $P_{ave}$ WV5 | $P_{ave}$ WV6 | $P_{ave}$ WV7 | $P_{ave}$ WV8 | $P_{ave}$ WV9 | $P_{ave}$ WV10 | ($P_{ave}$ WV1)^2 | ($P_{ave}$ WV2)^2 | ($P_{ave}$ WV3)^2 | ($P_{ave}$ WV4)^2 | ($P_{ave}$ WV5)^2 | ($P_{ave}$ WV6)^2 | ($P_{ave}$ WV7)^2 | ($P_{ave}$ WV8)^2 | ($P_{ave}$ WV9)^2 | ($P_{ave}$ WV10)^2 |
|---|---|---|---|---|---|---|---|---|---|---|---|---|---|---|---|---|---|---|---|---|---|
| 25 | 85.99 | 0.20 | -0.06 | -0.10 | 0.19 | 0.02 | 0.02 | 0.02 | 0.07 | -0.01 | 0.01 | 0.04 | 0.00 | 0.01 | 0.04 | 0.00 | 0.00 | 0.00 | 0.00 | 0.00 | 2.61E-005 |
| 25 | 85.99 | -0.10 | 0.02 | -0.06 | 0.28 | -0.03 | 0.06 | -0.05 | 0.02 | 0.02 | -0.02 | 0.01 | 0.00 | 0.00 | 0.08 | 0.00 | 0.00 | 0.00 | 0.00 | 0.00 | 0.00 |
| 25 | 75.19 | -0.05 | 0.04 | -0.09 | 0.27 | 0.03 | 0.04 | -0.01 | 0.01 | -0.00 | 0.07 | 0.00 | 0.00 | 0.01 | 0.07 | 0.00 | 0.00 | 4.82E-005 | 9.30E-005 | 8.96E-006 | 0.01 |
| 25 | 75.19 | -0.22 | -0.05 | -0.01 | 0.24 | -0.01 | -0.05 | 0.01 | -0.03 | 0.01 | 0.09 | 0.05 | 0.00 | 3.59E-005 | 0.06 | 8.03E-005 | 0.00 | 0.00 | 0.00 | 5.79E-005 | 0.01 |
| 25 | 75.19 | -0.10 | 0.03 | -0.11 | 0.24 | -0.01 | 0.05 | -0.04 | 0.02 | 0.02 | 0.01 | 0.01 | 0.00 | 0.01 | 0.06 | 0.00 | 0.00 | 0.00 | 0.00 | 0.00 | 0.00 |
| 25 | 132.44 | -0.03 | 0.00 | -0.15 | 0.29 | 0.02 | -0.02 | 0.02 | 0.08 | -0.04 | 0.04 | 0.00 | 1.25E-005 | 0.02 | 0.09 | 0.00 | 0.00 | 0.00 | 0.01 | 0.00 | 0.00 |
| 25 | 132.44 | -0.07 | 0.07 | -0.11 | 0.37 | -0.02 | 0.05 | 0.01 | -0.01 | 0.01 | -0.01 | 0.01 | 0.00 | 0.01 | 0.14 | 0.00 | 0.00 | 8.56E-005 | 3.04E-005 | 5.04E-005 | 3.80E-005 |
| 25 | 132.44 | -0.06 | -0.00 | -0.11 | 0.28 | -0.01 | -0.03 | -0.00 | 0.06 | 0.02 | 0.05 | 0.00 | 4.12E-007 | 0.01 | 0.08 | 5.60E-005 | 0.00 | 1.93E-005 | 0.00 | 0.00 | 0.00 |
| 25 | 132.44 | -0.00 | 0.03 | -0.11 | 0.29 | -0.01 | 0.01 | -0.01 | 0.02 | -0.01 | 0.04 | 1.22E-005 | 0.00 | 0.01 | 0.08 | 0.00 | 0.00 | 0.00 | 0.00 | 0.00 | 0.00 |
| 25 | 125.27 | 0.04 | -0.01 | -0.20 | 0.29 | 0.03 | 0.10 | 0.01 | 0.05 | -0.04 | -0.03 | 0.00 | 0.00 | 0.04 | 0.08 | 0.00 | 0.01 | 6.20E-005 | 0.00 | 0.00 | 0.00 |
| 25 | 80.95 | 0.06 | 0.05 | -0.13 | 0.31 | -0.01 | 0.01 | -0.04 | 0.03 | -0.02 | 0.01 | 0.00 | 0.00 | 0.02 | 0.10 | 6.31E-005 | 0.00 | 0.00 | 0.00 | 0.00 | 5.50E-005 |
| 25 | 132.44 | -0.17 | 0.05 | 0.00 | 0.33 | -0.06 | -0.00 | 0.01 | -0.07 | -0.04 | -0.01 | 0.03 | 0.00 | 6.91E-006 | 0.11 | 0.00 | 2.34E-005 | 0.00 | 0.00 | 0.00 | 6.15E-005 |
| 25 | 132.44 | -0.08 | 0.07 | -0.15 | 0.34 | 0.01 | 0.04 | 0.01 | -0.03 | -0.03 | 0.04 | 0.01 | 0.00 | 0.02 | 0.12 | 2.98E-005 | 0.00 | 0.00 | 0.00 | 0.00 | 0.00 |
| 25 | 17.94 | 0.01 | 0.04 | -0.13 | 0.26 | 0.02 | 0.03 | -0.04 | 0.02 | 0.02 | 0.00 | 4.12E-005 | 0.00 | 0.02 | 0.07 | 0.00 | 0.00 | 0.00 | 0.00 | 0.00 | 4.72E-006 |
| 50 | 88.15 | 0.20 | -0.06 | -0.10 | 0.19 | 0.02 | 0.02 | 0.02 | 0.07 | -0.01 | 0.01 | 0.04 | 0.00 | 0.01 | 0.04 | 0.00 | 0.00 | 0.00 | 0.00 | 0.00 | 2.61E-005 |
| 50 | 88.15 | -0.10 | 0.02 | -0.06 | 0.28 | -0.03 | 0.06 | -0.05 | 0.02 | 0.02 | -0.02 | 0.01 | 0.00 | 0.00 | 0.08 | 0.00 | 0.00 | 0.00 | 0.00 | 0.00 | 0.00 |
| 50 | 77.35 | -0.05 | 0.04 | -0.09 | 0.27 | 0.03 | 0.04 | -0.01 | 0.01 | -0.00 | 0.07 | 0.00 | 0.00 | 0.01 | 0.07 | 0.00 | 0.00 | 4.82E-005 | 9.30E-005 | 8.96E-006 | 0.01 |
| 50 | 77.35 | -0.22 | -0.05 | -0.01 | 0.24 | -0.01 | -0.05 | 0.01 | -0.03 | 0.01 | 0.09 | 0.05 | 0.00 | 3.59E-005 | 0.06 | 8.03E-005 | 0.00 | 0.00 | 0.00 | 5.79E-005 | 0.01 |
| 50 | 77.35 | -0.10 | 0.03 | -0.11 | 0.24 | -0.01 | 0.05 | -0.04 | 0.02 | 0.02 | 0.01 | 0.01 | 0.00 | 0.01 | 0.06 | 0.00 | 0.00 | 0.00 | 0.00 | 0.00 | 0.00 |
| 50 | 136.50 | -0.03 | 0.00 | -0.15 | 0.29 | 0.02 | -0.02 | 0.02 | 0.08 | -0.04 | 0.04 | 0.00 | 1.25E-005 | 0.02 | 0.09 | 0.00 | 0.00 | 0.00 | 0.01 | 0.00 | 0.00 |
| 50 | 136.50 | -0.07 | 0.07 | -0.11 | 0.37 | -0.02 | 0.05 | 0.01 | -0.01 | 0.01 | -0.01 | 0.01 | 0.00 | 0.01 | 0.14 | 0.00 | 0.00 | 8.56E-005 | 3.04E-005 | 5.04E-005 | 3.80E-005 |
| 50 | 136.50 | -0.06 | -0.00 | -0.11 | 0.28 | -0.01 | -0.03 | -0.00 | 0.06 | 0.02 | 0.05 | 0.00 | 4.12E-007 | 0.01 | 0.08 | 5.60E-005 | 0.00 | 1.93E-005 | 0.00 | 0.00 | 0.00 |
| 50 | 136.50 | -0.00 | 0.03 | -0.11 | 0.29 | -0.01 | 0.01 | -0.01 | 0.02 | -0.01 | 0.04 | 1.22E-005 | 0.00 | 0.01 | 0.08 | 0.00 | 0.00 | 0.00 | 0.00 | 0.00 | 0.00 |
| 50 | 129.22 | 0.04 | -0.01 | -0.20 | 0.29 | 0.03 | 0.10 | 0.01 | 0.05 | -0.04 | -0.03 | 0.00 | 0.00 | 0.04 | 0.08 | 0.00 | 0.01 | 6.20E-005 | 0.00 | 0.00 | 0.00 |
| 50 | 84.53 | 0.06 | 0.05 | -0.13 | 0.31 | -0.01 | 0.01 | -0.04 | 0.03 | -0.02 | 0.01 | 0.00 | 0.00 | 0.02 | 0.10 | 6.31E-005 | 0.00 | 0.00 | 0.00 | 0.00 | 5.50E-005 |
| 50 | 136.50 | -0.17 | 0.05 | 0.00 | 0.33 | -0.06 | -0.00 | 0.01 | -0.07 | -0.04 | -0.01 | 0.03 | 0.00 | 6.91E-006 | 0.11 | 0.00 | 2.34E-005 | 0.00 | 0.00 | 0.00 | 6.15E-005 |
| 50 | 136.50 | -0.08 | 0.07 | -0.15 | 0.34 | 0.01 | 0.04 | 0.01 | -0.03 | -0.03 | 0.04 | 0.01 | 0.00 | 0.02 | 0.12 | 2.98E-005 | 0.00 | 0.00 | 0.00 | 0.00 | 0.00 |
| 50 | 18.20 | 0.01 | 0.04 | -0.13 | 0.26 | 0.02 | 0.03 | -0.04 | 0.02 | 0.02 | 0.00 | 4.12E-005 | 0.00 | 0.02 | 0.07 | 0.00 | 0.00 | 0.00 | 0.00 | 0.00 | 4.72E-006 |
| 100 | 89.27 | 0.20 | -0.06 | -0.10 | 0.19 | 0.02 | 0.02 | 0.02 | 0.07 | -0.01 | 0.01 | 0.04 | 0.00 | 0.01 | 0.04 | 0.00 | 0.00 | 0.00 | 0.00 | 0.00 | 2.61E-005 |
| 100 | 89.27 | -0.10 | 0.02 | -0.06 | 0.28 | -0.03 | 0.06 | -0.05 | 0.02 | 0.02 | -0.02 | 0.01 | 0.00 | 0.00 | 0.08 | 0.00 | 0.00 | 0.00 | 0.00 | 0.00 | 0.00 |
| 100 | 78.47 | -0.05 | 0.04 | -0.09 | 0.27 | 0.03 | 0.04 | -0.01 | 0.01 | -0.00 | 0.07 | 0.00 | 0.00 | 0.01 | 0.07 | 0.00 | 0.00 | 4.82E-005 | 9.30E-005 | 8.96E-006 | 0.01 |
| 100 | 78.47 | -0.22 | -0.05 | -0.01 | 0.24 | -0.01 | -0.05 | 0.01 | -0.03 | 0.01 | 0.09 | 0.05 | 0.00 | 3.59E-005 | 0.06 | 8.03E-005 | 0.00 | 0.00 | 0.00 | 5.79E-005 | 0.01 |
| 100 | 78.47 | -0.10 | 0.03 | -0.11 | 0.24 | -0.01 | 0.05 | -0.04 | 0.02 | 0.02 | 0.01 | 0.01 | 0.00 | 0.01 | 0.06 | 0.00 | 0.00 | 0.00 | 0.00 | 0.00 | 0.00 |
| 100 | 138.59 | -0.03 | 0.00 | -0.15 | 0.29 | 0.02 | -0.02 | 0.02 | 0.08 | -0.04 | 0.04 | 0.00 | 1.25E-005 | 0.02 | 0.09 | 0.00 | 0.00 | 0.00 | 0.01 | 0.00 | 0.00 |
| 100 | 138.59 | -0.07 | 0.07 | -0.11 | 0.37 | -0.02 | 0.05 | 0.01 | -0.01 | 0.01 | -0.01 | 0.01 | 0.00 | 0.01 | 0.14 | 0.00 | 0.00 | 8.56E-005 | 3.04E-005 | 5.04E-005 | 3.80E-005 |
| 100 | 138.59 | -0.06 | -0.00 | -0.11 | 0.28 | -0.01 | -0.03 | -0.00 | 0.06 | 0.02 | 0.05 | 0.00 | 4.12E-007 | 0.01 | 0.08 | 5.60E-005 | 0.00 | 1.93E-005 | 0.00 | 0.00 | 0.00 |
| 100 | 138.59 | -0.00 | 0.03 | -0.11 | 0.29 | -0.01 | 0.01 | -0.01 | 0.02 | -0.01 | 0.04 | 1.22E-005 | 0.00 | 0.01 | 0.08 | 0.00 | 0.00 | 0.00 | 0.00 | 0.00 | 0.00 |
| 100 | 131.25 | 0.04 | -0.01 | -0.20 | 0.29 | 0.03 | 0.10 | 0.01 | 0.05 | -0.04 | -0.03 | 0.00 | 0.00 | 0.04 | 0.08 | 0.00 | 0.01 | 6.20E-005 | 0.00 | 0.00 | 0.00 |
| 100 | 86.38 | 0.06 | 0.05 | -0.13 | 0.31 | -0.01 | 0.01 | -0.04 | 0.03 | -0.02 | 0.01 | 0.00 | 0.00 | 0.02 | 0.10 | 6.31E-005 | 0.00 | 0.00 | 0.00 | 0.00 | 5.50E-005 |
| 100 | 138.59 | -0.17 | 0.05 | 0.00 | 0.33 | -0.06 | -0.00 | 0.01 | -0.07 | -0.04 | -0.01 | 0.03 | 0.00 | 6.91E-006 | 0.11 | 0.00 | 2.34E-005 | 0.00 | 0.00 | 0.00 | 6.15E-005 |
| 100 | 138.59 | -0.08 | 0.07 | -0.15 | 0.34 | 0.01 | 0.04 | 0.01 | -0.03 | -0.03 | 0.04 | 0.01 | 0.00 | 0.02 | 0.12 | 2.98E-005 | 0.00 | 0.00 | 0.00 | 0.00 | 0.00 |

| External glucose, mM | metabolic flux, mmol min $^{-1}$ | $P_{ave}$ WV1 | $P_{ave}$ WV2 | $P_{ave}$ WV3 | $P_{ave}$ WV4 | $P_{ave}$ WV5 | $P_{ave}$ WV6 | $P_{ave}$ WV7 | $P_{ave}$ WV8 | $P_{ave}$ WV9 | $P_{ave}$ WV10 | $(P_{ave}$ WV1$)^2$ | $(P_{ave}$ WV2$)^2$ | $(P_{ave}$ WV3$)^2$ | $(P_{ave}$ WV4$)^2$ | $(P_{ave}$ WV5$)^2$ | $(P_{ave}$ WV6$)^2$ | $(P_{ave}$ WV7$)^2$ | $(P_{ave}$ WV8$)^2$ | $(P_{ave}$ WV9$)^2$ | $(P_{ave}$ WV10$)^2$ |
|---|---|---|---|---|---|---|---|---|---|---|---|---|---|---|---|---|---|---|---|---|---|
| 100 | 18.34 | 0.01 | 0.04 | -0.13 | 0.26 | 0.02 | 0.03 | -0.04 | 0.02 | 0.02 | 0.00 | 4.12E-005 | 0.00 | 0.02 | 0.07 | 0.00 | 0.00 | 0.00 | 0.00 | 0.00 | 4.72E-006 |

[a] estimated by the Teusink's kinetic model as described in Material and Methods;
[b] in accordance with 10 VARIMAX-derived scales (Georgiev, 2009 [18]) which represent generalized numerical scores for physical-chemical and structural features of amino acids.

**Table S1-2**. The dataset used in multivariate analysis which comprises metabolic fluxes [a] and numerical vectors representing the average amino acid property [b] for each enzyme sequence.

| External glucose, mM | metabolic flux, mmol min $^{-1}$ | $P_{ave}$ WV1 | $P_{ave}$ WV2 | $P_{ave}$ WV3 | $P_{ave}$ WV4 | $P_{ave}$ WV5 | $P_{ave}$ WV6 | $P_{ave}$ WV7 | $P_{ave}$ WV8 | $P_{ave}$ WV9 | $P_{ave}$ WV10 | $(P_{ave}$ WV1$)^2$ | $(P_{ave}$ WV2$)^2$ | $(P_{ave}$ WV3$)^2$ | $(P_{ave}$ WV4$)^2$ | $(P_{ave}$ WV5$)^2$ | $(P_{ave}$ WV6$)^2$ | $(P_{ave}$ WV7$)^2$ | $(P_{ave}$ WV8$)^2$ | $(P_{ave}$ WV9$)^2$ | $(P_{ave}$ WV10$)^2$ |
|---|---|---|---|---|---|---|---|---|---|---|---|---|---|---|---|---|---|---|---|---|---|
| 25 | 0.84 | 20.09 | -5.87 | -9.85 | 19.15 | 1.64 | 3.87 | 1.56 | 6.93 | -1.10 | 0.51 | 405.66 | 34.44 | 104.19 | 366.91 | 2.68 | 26.78 | 3.83 | 40.91 | 1.21 | 0.48 |
| 25 | 49.36 | -9.75 | 2.10 | -5.61 | 28.44 | -3.18 | 5.59 | -4.90 | 1.72 | 2.10 | -2.10 | 95.03 | 4.42 | 31.46 | 809.06 | 10.09 | 31.29 | 23.97 | 2.96 | 4.42 | 4.40 |
| 25 | 28.58 | -4.88 | 3.73 | -8.79 | 26.64 | 3.46 | 3.76 | -0.69 | 0.96 | -0.30 | 7.23 | 23.81 | 13.94 | 77.31 | 709.47 | 11.95 | 14.13 | 0.48 | 0.93 | 0.09 | 52.27 |
| 25 | 28.58 | -21.96 | -5.43 | -0.60 | 23.99 | -0.90 | -4.83 | 1.43 | -3.30 | 0.76 | 8.75 | 482.11 | 29.54 | 0.36 | 575.31 | 0.80 | 23.33 | 2.04 | 10.91 | 0.58 | 76.56 |
| 25 | 28.58 | -9.68 | 3.35 | -10.89 | 23.67 | -1.14 | 4.95 | -3.85 | 1.54 | 2.14 | 1.10 | 93.70 | 11.22 | 118.51 | 560.48 | 1.31 | 24.54 | 14.79 | 2.38 | 4.56 | 1.21 |
| 25 | 24.27 | -6.01 | 4.08 | -12.24 | 33.83 | 4.30 | 3.52 | -1.30 | 2.96 | -1.45 | 2.43 | 36.09 | 16.67 | 149.81 | 1144.47 | 18.52 | 12.36 | 1.69 | 8.75 | 2.11 | 5.90 |
| 25 | 52.85 | -3.50 | 0.35 | -15.43 | 29.19 | 1.75 | -2.40 | 1.77 | 7.59 | -3.80 | 4.34 | 12.24 | 0.13 | 238.16 | 851.93 | 3.07 | 5.74 | 3.14 | 57.61 | 14.41 | 18.83 |
| 25 | 52.85 | -7.43 | 6.91 | -10.65 | 37.25 | -1.55 | 4.75 | 0.93 | -0.55 | 0.71 | -0.62 | 55.26 | 47.75 | 113.52 | 1387.26 | 2.41 | 22.58 | 0.86 | 0.30 | 0.50 | 0.38 |
| 25 | 52.85 | -5.93 | -0.06 | -11.28 | 28.26 | -0.75 | -3.02 | -0.44 | 6.44 | 1.65 | 5.44 | 35.11 | 0.00 | 127.27 | 798.65 | 0.56 | 9.13 | 0.19 | 41.50 | 2.72 | 29.58 |
| 25 | 52.85 | -0.35 | 2.64 | -11.10 | 29.12 | -1.32 | 1.33 | -1.03 | 1.85 | -1.32 | 4.20 | 0.12 | 6.98 | 123.16 | 848.14 | 1.74 | 1.77 | 1.06 | 3.42 | 1.74 | 17.67 |
| 25 | 48.54 | 3.63 | -1.37 | -20.07 | 28.59 | 2.61 | 10.28 | 0.79 | 5.02 | -4.16 | -3.36 | 13.20 | 1.88 | 402.82 | 817.43 | 6.81 | 105.67 | 0.62 | 25.24 | 17.28 | 11.32 |
| 25 | 6.98 | 5.63 | 4.56 | -13.44 | 31.18 | -0.79 | 1.31 | -3.53 | 3.21 | -2.12 | 0.74 | 31.67 | 20.81 | 180.75 | 972.18 | 0.63 | 1.73 | 12.49 | 10.28 | 4.51 | 0.55 |
| 25 | 0 | -15.60 | 7.22 | -12.60 | 26.79 | -8 | 5.60 | -0.25 | -1.05 | -2.26 | -0.08 | 243.45 | 52.16 | 158.85 | 717.96 | 64 | 31.32 | 0.06 | 1.10 | 5.13 | 0.01 |
| 25 | 20.78 | -6.66 | 2.03 | 0.74 | 26.41 | 1.46 | 1.43 | -4.46 | -1.30 | 5.58 | 4.65 | 44.32 | 4.14 | 0.55 | 697.27 | 2.13 | 2.05 | 19.87 | 1.70 | 31.17 | 21.67 |
| 25 | 4.31 | 0.64 | 3.78 | -12.84 | 25.91 | 2.31 | 3.46 | -3.82 | 1.70 | 1.69 | 0.22 | 0.41 | 14.27 | 164.90 | 671.32 | 5.35 | 11.94 | 14.60 | 2.88 | 2.84 | 0.05 |
| 50 | 0.84 | 20.09 | -5.87 | -9.85 | 19.15 | 1.64 | 3.87 | 1.56 | 6.93 | -1.10 | 0.51 | 405.66 | 34.44 | 104.19 | 366.91 | 2.68 | 26.78 | 3.83 | 40.91 | 1.21 | 0.48 |
| 50 | 49.38 | -9.75 | 2.10 | -5.61 | 28.44 | -3.18 | 5.59 | -4.90 | 1.72 | 2.10 | -2.10 | 95.03 | 4.42 | 31.46 | 809.06 | 10.09 | 31.29 | 23.97 | 2.96 | 4.42 | 4.40 |
| 50 | 28.60 | -4.88 | 3.73 | -8.79 | 26.64 | 3.46 | 3.76 | -0.69 | 0.96 | -0.30 | 7.23 | 23.81 | 13.94 | 77.31 | 709.47 | 11.95 | 14.13 | 0.48 | 0.93 | 0.09 | 52.27 |
| 50 | 28.60 | -21.96 | -5.43 | -0.60 | 23.99 | -0.90 | -4.83 | 1.43 | -3.30 | 0.76 | 8.75 | 482.11 | 29.54 | 0.36 | 575.31 | 0.80 | 23.33 | 2.04 | 10.91 | 0.58 | 76.56 |
| 50 | 28.60 | -9.68 | 3.35 | -10.89 | 23.67 | -1.14 | 4.95 | -3.85 | 1.54 | 2.14 | 1.10 | 93.70 | 11.22 | 118.51 | 560.48 | 1.31 | 24.54 | 14.79 | 2.38 | 4.56 | 1.21 |
| 50 | 24.28 | -6.01 | 4.08 | -12.24 | 33.83 | 4.30 | 3.52 | -1.30 | 2.96 | -1.45 | 2.43 | 36.09 | 16.67 | 149.81 | 1144.47 | 18.52 | 12.36 | 1.69 | 8.75 | 2.11 | 5.90 |
| 50 | 52.88 | -3.50 | 0.35 | -15.43 | 29.19 | 1.75 | -2.40 | 1.77 | 7.59 | -3.80 | 4.34 | 12.24 | 0.13 | 238.16 | 851.93 | 3.07 | 5.74 | 3.14 | 57.61 | 14.41 | 18.83 |
| 50 | 52.88 | -7.43 | 6.91 | -10.65 | 37.25 | -1.55 | 4.75 | 0.93 | -0.55 | 0.71 | -0.62 | 55.26 | 47.75 | 113.52 | 1387.26 | 2.41 | 22.58 | 0.86 | 0.30 | 0.50 | 0.38 |
| 50 | 52.88 | -5.93 | -0.06 | -11.28 | 28.26 | -0.75 | -3.02 | -0.44 | 6.44 | 1.65 | 5.44 | 35.11 | 0.00 | 127.27 | 798.65 | 0.56 | 9.13 | 0.19 | 41.50 | 2.72 | 29.58 |
| 50 | 52.88 | -0.35 | 2.64 | -11.10 | 29.12 | -1.32 | 1.33 | -1.03 | 1.85 | -1.32 | 4.20 | 0.12 | 6.98 | 123.16 | 848.14 | 1.74 | 1.77 | 1.06 | 3.42 | 1.74 | 17.67 |
| 50 | 48.56 | 3.63 | -1.37 | -20.07 | 28.59 | 2.61 | 10.28 | 0.79 | 5.02 | -4.16 | -3.36 | 13.20 | 1.88 | 402.82 | 817.43 | 6.81 | 105.67 | 0.62 | 25.24 | 17.28 | 11.32 |
| 50 | 6.98 | 5.63 | 4.56 | -13.44 | 31.18 | -0.79 | 1.31 | -3.53 | 3.21 | -2.12 | 0.74 | 31.67 | 20.81 | 180.75 | 972.18 | 0.63 | 1.73 | 12.49 | 10.28 | 4.51 | 0.55 |
| 50 | 0 | -15.60 | 7.22 | -12.60 | 26.79 | -8 | 5.60 | -0.25 | -1.05 | -2.26 | -0.08 | 243.45 | 52.16 | 158.85 | 717.96 | 64 | 31.32 | 0.06 | 1.10 | 5.13 | 0.01 |
| 50 | 20.79 | -6.66 | 2.03 | 0.74 | 26.41 | 1.46 | 1.43 | -4.46 | -1.30 | 5.58 | 4.65 | 44.32 | 4.14 | 0.55 | 697.27 | 2.13 | 2.05 | 19.87 | 1.70 | 31.17 | 21.67 |
| 50 | 4.32 | 0.64 | 3.78 | -12.84 | 25.91 | 2.31 | 3.46 | -3.82 | 1.70 | 1.69 | 0.22 | 0.41 | 14.27 | 164.90 | 671.32 | 5.35 | 11.94 | 14.60 | 2.88 | 2.84 | 0.05 |
| 100 | 0.84 | 20.09 | -5.87 | -9.85 | 19.15 | 1.64 | 3.87 | 1.56 | 6.93 | -1.10 | 0.51 | 405.66 | 34.44 | 104.19 | 366.91 | 2.68 | 26.78 | 3.83 | 40.91 | 1.21 | 0.48 |
| 100 | 49.39 | -9.75 | 2.10 | -5.61 | 28.44 | -3.18 | 5.59 | -4.90 | 1.72 | 2.10 | -2.10 | 95.03 | 4.42 | 31.46 | 809.06 | 10.09 | 31.29 | 23.97 | 2.96 | 4.42 | 4.40 |
| 100 | 28.60 | -4.88 | 3.73 | -8.79 | 26.64 | 3.46 | 3.76 | -0.69 | 0.96 | -0.30 | 7.23 | 23.81 | 13.94 | 77.31 | 709.47 | 11.95 | 14.13 | 0.48 | 0.93 | 0.09 | 52.27 |
| 100 | 28.60 | -21.96 | -5.43 | -0.60 | 23.99 | -0.90 | -4.83 | 1.43 | -3.30 | 0.76 | 8.75 | 482.11 | 29.54 | 0.36 | 575.31 | 0.80 | 23.33 | 2.04 | 10.91 | 0.58 | 76.56 |
| 100 | 28.60 | -9.68 | 3.35 | -10.89 | 23.67 | -1.14 | 4.95 | -3.85 | 1.54 | 2.14 | 1.10 | 93.70 | 11.22 | 118.51 | 560.48 | 1.31 | 24.54 | 14.79 | 2.38 | 4.56 | 1.21 |
| 100 | 24.28 | -6.01 | 4.08 | -12.24 | 33.83 | 4.30 | 3.52 | -1.30 | 2.96 | -1.45 | 2.43 | 36.09 | 16.67 | 149.81 | 1144.47 | 18.52 | 12.36 | 1.69 | 8.75 | 2.11 | 5.90 |
| 100 | 52.88 | -3.50 | 0.35 | -15.43 | 29.19 | 1.75 | -2.40 | 1.77 | 7.59 | -3.80 | 4.34 | 12.24 | 0.13 | 238.16 | 851.93 | 3.07 | 5.74 | 3.14 | 57.61 | 14.41 | 18.83 |
| 100 | 52.88 | -7.43 | 6.91 | -10.65 | 37.25 | -1.55 | 4.75 | 0.93 | -0.55 | 0.71 | -0.62 | 55.26 | 47.75 | 113.52 | 1387.26 | 2.41 | 22.58 | 0.86 | 0.30 | 0.50 | 0.38 |
| 100 | 52.88 | -5.93 | -0.06 | -11.28 | 28.26 | -0.75 | -3.02 | -0.44 | 6.44 | 1.65 | 5.44 | 35.11 | 0.00 | 127.27 | 798.65 | 0.56 | 9.13 | 0.19 | 41.50 | 2.72 | 29.58 |
| 100 | 52.88 | -0.35 | 2.64 | -11.10 | 29.12 | -1.32 | 1.33 | -1.03 | 1.85 | -1.32 | 4.20 | 0.12 | 6.98 | 123.16 | 848.14 | 1.74 | 1.77 | 1.06 | 3.42 | 1.74 | 17.67 |
| 100 | 48.56 | 3.63 | -1.37 | -20.07 | 28.59 | 2.61 | 10.28 | 0.79 | 5.02 | -4.16 | -3.36 | 13.20 | 1.88 | 402.82 | 817.43 | 6.81 | 105.67 | 0.62 | 25.24 | 17.28 | 11.32 |

| External glucose, mM | metabolic flux, mmol min $^{-1}$ | $P_{ave}$ WV1 | $P_{ave}$ WV2 | $P_{ave}$ WV3 | $P_{ave}$ WV4 | $P_{ave}$ WV5 | $P_{ave}$ WV6 | $P_{ave}$ WV7 | $P_{ave}$ WV8 | $P_{ave}$ WV9 | $P_{ave}$ WV10 | $(P_{ave}$ WV1)^2 | $(P_{ave}$ WV2)^2 | $(P_{ave}$ WV3)^2 | $(P_{ave}$ WV4)^2 | $(P_{ave}$ WV5)^2 | $(P_{ave}$ WV6)^2 | $(P_{ave}$ WV7)^2 | $(P_{ave}$ WV8)^2 | $(P_{ave}$ WV9)^2 | $(P_{ave}$ WV10)^2 |
|---|---|---|---|---|---|---|---|---|---|---|---|---|---|---|---|---|---|---|---|---|---|
| 100 | 6.98 | 5.63 | 4.56 | -13.44 | 31.18 | -0.79 | 1.31 | -3.53 | 3.21 | -2.12 | 0.74 | 31.67 | 20.81 | 180.75 | 972.18 | 0.63 | 1.73 | 12.49 | 10.28 | 4.51 | 0.55 |
| 100 | -5.68E-014 | -15.60 | 7.22 | -12.60 | 26.79 | -8 | 5.60 | -0.25 | -1.05 | -2.26 | -0.08 | 243.45 | 52.16 | 158.85 | 717.96 | 64 | 31.32 | 0.06 | 1.10 | 5.13 | 0.01 |
| 100 | 20.79 | -6.66 | 2.03 | 0.74 | 26.41 | 1.46 | 1.43 | -4.46 | -1.30 | 5.58 | 4.65 | 44.32 | 4.14 | 0.55 | 697.27 | 2.13 | 2.05 | 19.87 | 1.70 | 31.17 | 21.67 |
| 100 | 4.32 | 0.64 | 3.78 | -12.84 | 25.91 | 2.31 | 3.46 | -3.82 | 1.70 | 1.69 | 0.22 | 0.41 | 14.27 | 164.90 | 671.32 | 5.35 | 11.94 | 14.60 | 2.88 | 2.84 | 0.05 |

[a] estimated by the Hynne's kinetic model as described in Material and Methods;
[b] in accordance with 10 VARIMAX-derived scales (Georgiev, 2009 [18]) which represent generalized numerical scores for physical-chemical and structural features of amino acids.

**Table S1-3**. The dataset used in multivariate analysis which comprises metabolic fluxes [a] and numerical vectors representing the average amino acid property [b] for each enzyme sequence.

| External glucose, mM | metabolic flux, mmol min $^{-1}$ | $P_{ave}$ WV1-1 | $P_{ave}$ WV1-2 | $P_{ave}$ WV1-3 | $(P_{ave}$ WV1-1)^2 | $(P_{ave}$ WV1-2)^2 | $(P_{ave}$ WV1-3)^2 | $P_{ave}$ WV2-1 | $P_{ave}$ WV2-2 | $P_{ave}$ WV2-3 | $(P_{ave}$ WV2-1)^2 | $(P_{ave}$ WV2-2)^2 | $(P_{ave}$ WV2-3)^2 | $P_{ave}$ WV3-1 | $P_{ave}$ WV3-2 | $P_{ave}$ WV3-3 | $(P_{ave}$ WV3-1)^2 | $(P_{ave}$ WV3-2)^2 | $(P_{ave}$ WV3-3)^2 | $P_{ave}$ WV5-1 | $P_{ave}$ WV5-2 | $P_{ave}$ WV5-3 | $(P_{ave}$ WV5-1)^2 | $(P_{ave}$ WV5-2)^2 | $(P_{ave}$ WV5-3)^2 | $P_{ave}$ WV6-1 | $P_{ave}$ WV6-2 | $P_{ave}$ WV6-3 | $(P_{ave}$ WV6-1)^2 | $(P_{ave}$ WV6-2)^2 | $(P_{ave}$ WV6-3)^2 | $P_{ave}$ WV7-1 | $P_{ave}$ WV7-2 | $P_{ave}$ WV 7-3 | $(P_{ave}$ WV7-1)^2 | $(P_{ave}$ WV7-2)^2 | $(P_{ave}$ WV7-3)^2 |
|---|---|---|---|---|---|---|---|---|---|---|---|---|---|---|---|---|---|---|---|---|---|---|---|---|---|---|---|---|---|---|---|---|---|---|---|---|---|
| 25 | 85.99 | 10.30 | 33.63 | 0.75 | 106.15 | 1130.78 | 0.56 | 0.98 | 0.99 | 0.99 | 0.97 | 0.97 | 0.99 | 143.74 | 138.34 | 3.30 | 20660.76 | 19137.84 | 10.89 | 8.02 | 1.56 | 0.96 | 64.28 | 2.44 | 0.93 | 0.99 | 11.68 | 1.21 | 0.99 | 136.45 | 1.47 | 0.00 | 5.96 | 1.05 | 1.00E-006 | 35.52 | 1.09 |
| 25 | 85.99 | -15.80 | 7.82 | 0.72 | 249.55 | 61.20 | 0.52 | 1.02 | 1.03 | 1.04 | 1.03 | 1.06 | 1.07 | 142.46 | 137.45 | 3.25 | 20295.82 | 18892.31 | 10.55 | 7.92 | 1.72 | 1.04 | 62.79 | 2.95 | 1.07 | 1.01 | 11.19 | 1.18 | 1.02 | 125.29 | 1.40 | -0.03 | 5.96 | 1.04 | 0.00 | 35.55 | 1.09 |
| 25 | 75.19 | -9.69 | 11.13 | 0.72 | 93.80 | 123.88 | 0.52 | 1.02 | 1.03 | 1.04 | 1.04 | 1.06 | 1.08 | 141.89 | 136.94 | 3.21 | 20132.26 | 18752.29 | 10.32 | 8.12 | 1.42 | 0.99 | 65.96 | 2.01 | 0.99 | 1.01 | 11.25 | 1.21 | 1.03 | 126.55 | 1.46 | -0.02 | 5.94 | 1.06 | 0.00 | 35.29 | 1.13 |
| 25 | 75.19 | -25.82 | -2.95 | 0.71 | 666.52 | 8.68 | 0.50 | 0.99 | 1.00 | 0.99 | 0.97 | 1.00 | 0.98 | 144.43 | 138.74 | 3.36 | 20860.80 | 19248.18 | 11.29 | 7.86 | 1.81 | 1.04 | 61.72 | 3.26 | 1.08 | 0.97 | 11.60 | 1.19 | 0.93 | 134.59 | 1.42 | 0.01 | 6.08 | 1.10 | 6.40E-005 | 36.93 | 1.22 |
| 25 | 75.19 | -14.64 | 7.29 | 0.72 | 214.45 | 53.17 | 0.52 | 1.02 | 1.03 | 1.04 | 1.04 | 1.07 | 1.08 | 141.39 | 135.89 | 3.17 | 19991.67 | 18465.66 | 10.07 | 7.98 | 1.63 | 1.02 | 63.70 | 2.66 | 1.04 | 1.03 | 11.17 | 1.19 | 1.05 | 124.80 | 1.41 | -0.03 | 5.94 | 1.05 | 0.00 | 35.32 | 1.11 |
| 25 | 132.44 | -8.11 | 10.02 | 0.72 | 65.76 | 100.46 | 0.52 | 1.00 | 1.01 | 1.01 | 1.00 | 1.02 | 1.03 | 138.11 | 133.47 | 3.03 | 19075.73 | 17814.16 | 9.18 | 7.99 | 1.53 | 1.00 | 63.88 | 2.34 | 1.00 | 0.98 | 11.43 | 1.10 | 0.95 | 130.62 | 1.20 | 0.01 | 6.04 | 1.06 | 3.60E-005 | 36.47 | 1.12 |
| 25 | 132.44 | -12.91 | 7.83 | 0.72 | 166.75 | 61.25 | 0.52 | 1.03 | 1.04 | 1.05 | 1.06 | 1.08 | 1.11 | 138.53 | 134.52 | 3.05 | 19191.12 | 18096.73 | 9.33 | 8.01 | 1.57 | 1.03 | 64.18 | 2.47 | 1.05 | 1.01 | 11.02 | 1.14 | 1.03 | 121.39 | 1.29 | 0 | 6.06 | 1.07 | 0 | 36.77 | 1.14 |
| 25 | 132.44 | -10.64 | 9.26 | 0.72 | 113.17 | 85.67 | 0.52 | 1.01 | 1.02 | 1.02 | 1.01 | 1.03 | 1.04 | 140.57 | 135.74 | 3.14 | 19760.88 | 18423.99 | 9.84 | 7.94 | 1.67 | 1.02 | 63.07 | 2.78 | 1.04 | 0.97 | 11.67 | 1.12 | 0.94 | 136.24 | 1.26 | 0.00 | 6.05 | 1.06 | 4.00E-006 | 36.58 | 1.12 |
| 25 | 132.44 | -6.03 | 14.37 | 0.73 | 36.39 | 206.50 | 0.53 | 1.01 | 1.02 | 1.03 | 1.03 | 1.04 | 1.06 | 140.22 | 135.50 | 3.12 | 19660.75 | 18360.71 | 9.76 | 7.95 | 1.62 | 1.01 | 63.17 | 2.62 | 1.02 | 0.98 | 11.33 | 1.15 | 0.97 | 128.27 | 1.33 | -0.02 | 5.94 | 1.05 | 0.00 | 35.24 | 1.09 |
| 25 | 125.27 | -3.21 | 16.31 | 0.73 | 10.30 | 265.95 | 0.53 | 0.99 | 1.00 | 1.00 | 0.99 | 1.01 | 1.01 | 135.68 | 130.95 | 2.92 | 18410.12 | 17148.37 | 8.55 | 8.03 | 1.52 | 0.99 | 64.49 | 2.32 | 0.97 | 1.06 | 10.79 | 1.21 | 1.13 | 116.47 | 1.47 | -0.01 | 5.98 | 1.04 | 0.00 | 35.79 | 1.07 |
| 25 | 80.95 | 0.15 | 19.61 | 0.73 | 0.02 | 384.36 | 0.54 | 1.02 | 1.03 | 1.04 | 1.05 | 1.05 | 1.09 | 139.44 | 135.03 | 3.09 | 19443.65 | 18232.10 | 9.56 | 8.01 | 1.55 | 1.00 | 64.11 | 2.40 | 1.01 | 0.97 | 11.31 | 1.14 | 0.94 | 128.02 | 1.29 | -0.04 | 5.90 | 1.03 | 0.00 | 34.77 | 1.07 |
| 25 | 132.44 | -22.98 | 1.13 | 0.71 | 528.26 | 1.27 | 0.50 | 1.03 | 1.04 | 1.05 | 1.06 | 1.08 | 1.09 | 143.55 | 138.85 | 3.29 | 20605.20 | 19278.66 | 10.85 | 7.82 | 1.85 | 1.06 | 61.14 | 3.42 | 1.13 | 0.97 | 11.21 | 1.11 | 0.94 | 125.70 | 1.24 | 0.01 | 6.14 | 1.09 | 0.00 | 37.65 | 1.19 |
| 25 | 132.44 | -11.57 | 6.71 | 0.72 | 133.77 | 44.98 | 0.52 | 1.03 | 1.04 | 1.06 | 1.06 | 1.09 | 1.12 | 137.68 | 133.28 | 2.98 | 18954.54 | 17763.56 | 8.90 | 8.09 | 1.49 | 1.02 | 65.50 | 2.22 | 1.04 | 1.01 | 10.85 | 1.15 | 1.02 | 117.62 | 1.32 | -0.01 | 6.03 | 1.08 | 0.00 | 36.41 | 1.16 |
| 25 | 17.94 | -5.24 | 16.95 | 0.73 | 27.43 | 287.30 | 0.53 | 1.02 | 1.03 | 1.04 | 1.04 | 1.06 | 1.07 | 140.23 | 135.40 | 3.15 | 19665.01 | 18333.08 | 9.92 | 8.05 | 1.52 | 1.00 | 64.83 | 2.31 | 0.99 | 1.01 | 11.25 | 1.18 | 1.01 | 126.55 | 1.40 | -0.04 | 5.91 | 1.04 | 0.00 | 34.91 | 1.08 |
| 50 | 88.15 | 10.30 | 33.63 | 0.75 | 106.15 | 1130.78 | 0.56 | 0.98 | 0.99 | 0.99 | 0.97 | 0.97 | 0.99 | 143.74 | 138.34 | 3.30 | 20660.76 | 19137.84 | 10.89 | 8.02 | 1.56 | 0.96 | 64.28 | 2.44 | 0.93 | 0.99 | 11.68 | 1.21 | 0.99 | 136.45 | 1.47 | 0.00 | 5.96 | 1.05 | 1.00E-006 | 35.52 | 1.09 |
| 50 | 88.15 | -15.80 | 7.82 | 0.72 | 249.55 | 61.20 | 0.52 | 1.02 | 1.03 | 1.04 | 1.03 | 1.06 | 1.07 | 142.46 | 137.45 | 3.25 | 20295.82 | 18892.31 | 10.55 | 7.92 | 1.72 | 1.04 | 62.79 | 2.95 | 1.07 | 1.01 | 11.19 | 1.18 | 1.02 | 125.29 | 1.40 | -0.03 | 5.96 | 1.04 | 0.00 | 35.55 | 1.09 |
| 50 | 77.35 | -9.69 | 11.13 | 0.72 | 93.80 | 123.88 | 0.52 | 1.02 | 1.03 | 1.04 | 1.04 | 1.06 | 1.08 | 141.89 | 136.94 | 3.21 | 20132.26 | 18752.29 | 10.32 | 8.12 | 1.42 | 0.99 | 65.96 | 2.01 | 0.99 | 1.01 | 11.25 | 1.21 | 1.03 | 126.55 | 1.46 | -0.02 | 5.94 | 1.06 | 0.00 | 35.29 | 1.13 |
| 50 | 77.35 | -25.82 | -2.95 | 0.71 | 666.52 | 8.68 | 0.50 | 0.99 | 1.00 | 0.99 | 0.97 | 1.00 | 0.98 | 144.43 | 138.74 | 3.36 | 20860.80 | 19248.18 | 11.29 | 7.86 | 1.81 | 1.04 | 61.72 | 3.26 | 1.08 | 0.97 | 11.60 | 1.19 | 0.93 | 134.59 | 1.42 | 0.01 | 6.08 | 1.10 | 6.40E-005 | 36.93 | 1.22 |
| 50 | 77.35 | -14.64 | 7.29 | 0.72 | 214.45 | 53.17 | 0.52 | 1.02 | 1.03 | 1.04 | 1.04 | 1.07 | 1.08 | 141.39 | 135.89 | 3.17 | 19991.67 | 18465.66 | 10.07 | 7.98 | 1.63 | 1.02 | 63.70 | 2.66 | 1.04 | 1.03 | 11.17 | 1.19 | 1.05 | 124.80 | 1.41 | -0.03 | 5.94 | 1.05 | 0.00 | 35.32 | 1.11 |
| 50 | 136.50 | -8.11 | 10.02 | 0.72 | 65.76 | 100.46 | 0.52 | 1.00 | 1.01 | 1.01 | 1.00 | 1.02 | 1.03 | 138.11 | 133.47 | 3.03 | 19075.73 | 17814.16 | 9.18 | 7.99 | 1.53 | 1.00 | 63.88 | 2.34 | 1.00 | 0.98 | 11.43 | 1.10 | 0.95 | 130.62 | 1.20 | 0.01 | 6.04 | 1.06 | 3.60E-005 | 36.47 | 1.12 |
| 50 | 136.50 | -12.91 | 7.83 | 0.72 | 166.75 | 61.25 | 0.52 | 1.03 | 1.04 | 1.05 | 1.06 | 1.08 | 1.11 | 138.53 | 134.52 | 3.05 | 19191.12 | 18096.73 | 9.33 | 8.01 | 1.57 | 1.03 | 64.18 | 2.47 | 1.05 | 1.01 | 11.02 | 1.14 | 1.03 | 121.39 | 1.29 | 0 | 6.06 | 1.07 | 0 | 36.77 | 1.14 |
| 50 | 136.50 | -10.64 | 9.26 | 0.72 | 113.17 | 85.67 | 0.52 | 1.01 | 1.02 | 1.02 | 1.01 | 1.03 | 1.04 | 140.57 | 135.74 | 3.14 | 19760.88 | 18423.99 | 9.84 | 7.94 | 1.67 | 1.02 | 63.07 | 2.78 | 1.04 | 0.97 | 11.67 | 1.12 | 0.94 | 136.24 | 1.26 | 0.00 | 6.05 | 1.06 | 4.00E-006 | 36.58 | 1.12 |
| 50 | 136.50 | -6.03 | 14.37 | 0.73 | 36.39 | 206.50 | 0.53 | 1.01 | 1.02 | 1.03 | 1.03 | 1.04 | 1.06 | 140.22 | 135.50 | 3.12 | 19660.75 | 18360.71 | 9.76 | 7.95 | 1.62 | 1.01 | 63.17 | 2.62 | 1.02 | 0.98 | 11.33 | 1.15 | 0.97 | 128.27 | 1.33 | -0.02 | 5.94 | 1.05 | 0.00 | 35.24 | 1.09 |
| 50 | 129.22 | -3.21 | 16.31 | 0.73 | 10.30 | 265.95 | 0.53 | 0.99 | 1.00 | 1.00 | 0.99 | 1.01 | 1.01 | 135.68 | 130.95 | 2.92 | 18410.12 | 17148.37 | 8.55 | 8.03 | 1.52 | 0.99 | 64.49 | 2.32 | 0.97 | 1.06 | 10.79 | 1.21 | 1.13 | 116.47 | 1.47 | -0.01 | 5.98 | 1.04 | 0.00 | 35.79 | 1.07 |
| 50 | 84.53 | 0.15 | 19.61 | 0.73 | 0.02 | 384.36 | 0.54 | 1.02 | 1.03 | 1.04 | 1.05 | 1.05 | 1.09 | 139.44 | 135.03 | 3.09 | 19443.65 | 18232.10 | 9.56 | 8.01 | 1.55 | 1.00 | 64.11 | 2.40 | 1.01 | 0.97 | 11.31 | 1.14 | 0.94 | 128.02 | 1.29 | -0.04 | 5.90 | 1.03 | 0.00 | 34.77 | 1.07 |
| 50 | 136.50 | -22.98 | 1.13 | 0.71 | 528.26 | 1.27 | 0.50 | 1.03 | 1.04 | 1.05 | 1.06 | 1.08 | 1.09 | 143.55 | 138.85 | 3.29 | 20605.20 | 19278.66 | 10.85 | 7.82 | 1.85 | 1.06 | 61.14 | 3.42 | 1.13 | 0.97 | 11.21 | 1.11 | 0.94 | 125.70 | 1.24 | 0.01 | 6.14 | 1.09 | 0.00 | 37.65 | 1.19 |
| 50 | 136.50 | -11.57 | 6.71 | 0.72 | 133.77 | 44.98 | 0.52 | 1.03 | 1.04 | 1.06 | 1.06 | 1.09 | 1.12 | 137.68 | 133.28 | 2.98 | 18954.54 | 17763.56 | 8.90 | 8.09 | 1.49 | 1.02 | 65.50 | 2.22 | 1.04 | 1.01 | 10.85 | 1.15 | 1.02 | 117.62 | 1.32 | -0.01 | 6.03 | 1.08 | 0.00 | 36.41 | 1.16 |
| 50 | 18.20 | -5.24 | 16.95 | 0.73 | 27.43 | 287.30 | 0.53 | 1.02 | 1.03 | 1.04 | 1.04 | 1.06 | 1.07 | 140.23 | 135.40 | 3.15 | 19665.01 | 18333.08 | 9.92 | 8.05 | 1.52 | 1.00 | 64.83 | 2.31 | 0.99 | 1.01 | 11.25 | 1.18 | 1.01 | 126.55 | 1.40 | -0.04 | 5.91 | 1.04 | 0.00 | 34.91 | 1.08 |
| 100 | 89.27 | 10.30 | 33.63 | 0.75 | 106.15 | 1130.78 | 0.56 | 0.98 | 0.99 | 0.99 | 0.97 | 0.97 | 0.99 | 143.74 | 138.34 | 3.30 | 20660.76 | 19137.84 | 10.89 | 8.02 | 1.56 | 0.96 | 64.28 | 2.44 | 0.93 | 0.99 | 11.68 | 1.21 | 0.99 | 136.45 | 1.47 | 0.00 | 5.96 | 1.05 | 1.00E-006 | 35.52 | 1.09 |
| 100 | 89.27 | -15.80 | 7.82 | 0.72 | 249.55 | 61.20 | 0.52 | 1.02 | 1.03 | 1.04 | 1.03 | 1.06 | 1.07 | 142.46 | 137.45 | 3.25 | 20295.82 | 18892.31 | 10.55 | 7.92 | 1.72 | 1.04 | 62.79 | 2.95 | 1.07 | 1.01 | 11.19 | 1.18 | 1.02 | 125.29 | 1.40 | -0.03 | 5.96 | 1.04 | 0.00 | 35.55 | 1.09 |
| 100 | 78.47 | -9.69 | 11.13 | 0.72 | 93.80 | 123.88 | 0.52 | 1.02 | 1.03 | 1.04 | 1.04 | 1.06 | 1.08 | 141.89 | 136.94 | 3.21 | 20132.26 | 18752.29 | 10.32 | 8.12 | 1.42 | 0.99 | 65.96 | 2.01 | 0.99 | 1.01 | 11.25 | 1.21 | 1.03 | 126.55 | 1.46 | -0.02 | 5.94 | 1.06 | 0.00 | 35.29 | 1.13 |
| 100 | 78.47 | -25.82 | -2.95 | 0.71 | 666.52 | 8.68 | 0.50 | 0.99 | 1.00 | 0.99 | 0.97 | 1.00 | 0.98 | 144.43 | 138.74 | 3.36 | 20860.80 | 19248.18 | 11.29 | 7.86 | 1.81 | 1.04 | 61.72 | 3.26 | 1.08 | 0.97 | 11.60 | 1.19 | 0.93 | 134.59 | 1.42 | 0.01 | 6.08 | 1.10 | 6.40E-005 | 36.93 | 1.22 |
| 100 | 78.47 | -14.64 | 7.29 | 0.72 | 214.45 | 53.17 | 0.52 | 1.02 | 1.03 | 1.04 | 1.04 | 1.07 | 1.08 | 141.39 | 135.89 | 3.17 | 19991.67 | 18465.66 | 10.07 | 7.98 | 1.63 | 1.02 | 63.70 | 2.66 | 1.04 | 1.03 | 11.17 | 1.19 | 1.05 | 124.80 | 1.41 | -0.03 | 5.94 | 1.05 | 0.00 | 35.32 | 1.11 |
| 100 | 138.59 | -8.11 | 10.02 | 0.72 | 65.76 | 100.46 | 0.52 | 1.00 | 1.01 | 1.01 | 1.00 | 1.02 | 1.03 | 138.11 | 133.47 | 3.03 | 19075.73 | 17814.16 | 9.18 | 7.99 | 1.53 | 1.00 | 63.88 | 2.34 | 1.00 | 0.98 | 11.43 | 1.10 | 0.95 | 130.62 | 1.20 | 0.01 | 6.04 | 1.06 | 3.60E-005 | 36.47 | 1.12 |
| 100 | 138.59 | -12.91 | 7.83 | 0.72 | 166.75 | 61.25 | 0.52 | 1.03 | 1.04 | 1.05 | 1.06 | 1.08 | 1.11 | 138.53 | 134.52 | 3.05 | 19191.12 | 18096.73 | 9.33 | 8.01 | 1.57 | 1.03 | 64.18 | 2.47 | 1.05 | 1.01 | 11.02 | 1.14 | 1.03 | 121.39 | 1.29 | 0 | 6.06 | 1.07 | 0 | 36.77 | 1.14 |
| 100 | 138.59 | -10.64 | 9.26 | 0.72 | 113.17 | 85.67 | 0.52 | 1.01 | 1.02 | 1.02 | 1.01 | 1.03 | 1.04 | 140.57 | 135.74 | 3.14 | 19760.88 | 18423.99 | 9.84 | 7.94 | 1.67 | 1.02 | 63.07 | 2.78 | 1.04 | 0.97 | 11.67 | 1.12 | 0.94 | 136.24 | 1.26 | 0.00 | 6.05 | 1.06 | 4.00E-006 | 36.58 | 1.12 |
| 100 | 138.59 | -6.03 | 14.37 | 0.73 | 36.39 | 206.50 | 0.53 | 1.01 | 1.02 | 1.03 | 1.03 | 1.04 | 1.06 | 140.22 | 135.50 | 3.12 | 19660.75 | 18360.71 | 9.76 | 7.95 | 1.62 | 1.01 | 63.17 | 2.62 | 1.02 | 0.98 | 11.33 | 1.15 | 0.97 | 128.27 | 1.33 | -0.02 | 5.94 | 1.05 | 0.00 | 35.24 | 1.09 |
| 100 | 131.25 | -3.21 | 16.31 | 0.73 | 10.30 | 265.95 | 0.53 | 0.99 | 1.00 | 1.00 | 0.99 | 1.01 | 1.01 | 135.68 | 130.95 | 2.92 | 18410.12 | 17148.37 | 8.55 | 8.03 | 1.52 | 0.99 | 64.49 | 2.32 | 0.97 | 1.06 | 10.79 | 1.21 | 1.13 | 116.47 | 1.47 | -0.01 | 5.98 | 1.04 | 0.00 | 35.79 | 1.07 |

| External glucose, mM | metabolic flux, mmol min$^{-1}$ | $P_{ave}$ WV1-1 | $P_{ave}$ WV1-2 | $P_{ave}$ WV1-3 | ($P_{ave}$ WV1-1)^2 | ($P_{ave}$ WV1-2)^2 | ($P_{ave}$ WV1-3)^2 | $P_{ave}$ WV2-1 | $P_{ave}$ WV2-2 | $P_{ave}$ WV2-3 | ($P_{ave}$ WV2-1)^2 | ($P_{ave}$ WV2-2)^2 | ($P_{ave}$ WV2-3)^2 | $P_{ave}$ WV3-1 | $P_{ave}$ WV3-2 | $P_{ave}$ WV3-3 | ($P_{ave}$ WV3-1)^2 | ($P_{ave}$ WV3-2)^2 | ($P_{ave}$ WV3-3)^2 | $P_{ave}$ WV5-1 | $P_{ave}$ WV5-2 | $P_{ave}$ WV5-3 | ($P_{ave}$ WV5-1)^2 | ($P_{ave}$ WV5-2)^2 | ($P_{ave}$ WV5-3)^2 | $P_{ave}$ WV6-1 | $P_{ave}$ WV6-2 | $P_{ave}$ WV6-3 | ($P_{ave}$ WV6-1)^2 | ($P_{ave}$ WV6-2)^2 | ($P_{ave}$ WV6-3)^2 | $P_{ave}$ WV7-1 | $P_{ave}$ WV7-2 | $P_{ave}$ WV 7-3 | ($P_{ave}$ WV7-1)^2 | ($P_{ave}$ WV7-2)^2 | ($P_{ave}$ WV7-3)^2 |
|---|---|---|---|---|---|---|---|---|---|---|---|---|---|---|---|---|---|---|---|---|---|---|---|---|---|---|---|---|---|---|---|---|---|---|---|---|---|
| 100 | 86.38 | 0.15 | 19.61 | 0.73 | 0.02 | 384.36 | 0.54 | 1.02 | 1.03 | 1.04 | 1.05 | 1.05 | 1.09 | 139.44 | 135.03 | 3.09 | 19443.65 | 18232.10 | 9.56 | 8.01 | 1.55 | 1.00 | 64.11 | 2.40 | 1.01 | 0.97 | 11.31 | 1.14 | 0.94 | 128.02 | 1.29 | -0.04 | 5.90 | 1.03 | 0.00 | 34.77 | 1.07 |
| 100 | 138.59 | -22.98 | 1.13 | 0.71 | 528.26 | 1.27 | 0.50 | 1.03 | 1.04 | 1.05 | 1.06 | 1.08 | 1.09 | 143.55 | 138.85 | 3.29 | 20605.20 | 19278.66 | 10.85 | 7.82 | 1.85 | 1.06 | 61.14 | 3.42 | 1.13 | 0.97 | 11.21 | 1.11 | 0.94 | 125.70 | 1.24 | 0.01 | 6.14 | 1.09 | 0.00 | 37.65 | 1.19 |
| 100 | 138.59 | -11.57 | 6.71 | 0.72 | 133.77 | 44.98 | 0.52 | 1.03 | 1.04 | 1.06 | 1.06 | 1.09 | 1.12 | 137.68 | 133.28 | 2.98 | 18954.54 | 17763.56 | 8.90 | 8.09 | 1.49 | 1.02 | 65.50 | 2.22 | 1.04 | 1.01 | 10.85 | 1.15 | 1.02 | 117.62 | 1.32 | -0.01 | 6.03 | 1.08 | 0.00 | 36.41 | 1.16 |
| 100 | 18.34 | -5.24 | 16.95 | 0.73 | 27.43 | 287.30 | 0.53 | 1.02 | 1.03 | 1.04 | 1.04 | 1.06 | 1.07 | 140.23 | 135.40 | 3.15 | 19665.01 | 18333.08 | 9.92 | 8.05 | 1.52 | 1.00 | 64.83 | 2.31 | 0.99 | 1.01 | 11.25 | 1.18 | 1.01 | 126.55 | 1.40 | -0.04 | 5.91 | 1.04 | 0.00 | 34.91 | 1.08 |

[a] estimated by the Teusink's kinetic model as described in Material and Methods;
[b] in accordance with 10 VARIMAX-derived scales (Georgiev, 2009 [18]) which represent generalized numerical scores for physical-chemical and structural features of amino acids.

**Table S1-4**. The dataset used in multivariate analysis which comprises metabolic fluxes [a] and numerical vectors representing the average amino acid property [b] for each enzyme sequence.

| External glucose, mM | metabolic flux, mmol min$^{-1}$ | $P_{ave}$ WV1-1 | $P_{ave}$ WV1-2 | $P_{ave}$ WV1-3 | ($P_{ave}$ WV1-1)^2 | ($P_{ave}$ WV1-2)^2 | ($P_{ave}$ WV 1-3)^2 | $P_{ave}$ WV2-1 | $P_{ave}$ WV2-2 | $P_{ave}$ WV2-3 | ($P_{ave}$ WV2-1)^2 | ($P_{ave}$ WV2-2)^2 | ($P_{ave}$ WV2-3)^2 | $P_{ave}$ WV3-1 | $P_{ave}$ WV3-2 | $P_{ave}$ WV3-3 | ($P_{ave}$ WV3-1)^2 | ($P_{ave}$ WV3-2)^2 | ($P_{ave}$ WV3-3)^2 | $P_{ave}$ WV5-1 | $P_{ave}$ WV5-2 | $P_{ave}$ WV5-3 | ($P_{ave}$ WV5-1)^2 | ($P_{ave}$ WV5-2)^2 | ($P_{ave}$ WV5-3)^2 | $P_{ave}$ WV6-1 | $P_{ave}$ WV6-2 | $P_{ave}$ WV6-3 | ($P_{ave}$ WV6-1)^2 | ($P_{ave}$ WV6-2)^2 | ($P_{ave}$ WV6-3)^2 | $P_{ave}$ WV7-1 | $P_{ave}$ WV 7-2 | $P_{ave}$ WV 7-3 | ($P_{ave}$ WV7-1)^2 | ($P_{ave}$ WV7-2)^2 | ($P_{ave}$ WV7-3)^2 |
|---|---|---|---|---|---|---|---|---|---|---|---|---|---|---|---|---|---|---|---|---|---|---|---|---|---|---|---|---|---|---|---|---|---|---|---|---|---|
| 25 | 0.84 | 10.44 | 33.56 | 0.75 | 109.01 | 1126.01 | 0.56 | 0.98 | 0.99 | 0.99 | 0.97 | 0.97 | 0.99 | 143.07 | 137.67 | 3.34 | 20467.79 | 18951.84 | 11.18 | 8.02 | 1.56 | 0.96 | 64.28 | 2.44 | 0.93 | 0.99 | 11.68 | 1.21 | 0.99 | 136.45 | 1.47 | 0.00 | 5.96 | 1.05 | 1.00E-006 | 35.52 | 1.09 |
| 25 | 49.36 | -15.80 | 7.82 | 0.72 | 249.55 | 61.20 | 0.52 | 1.02 | 1.03 | 1.04 | 1.03 | 1.06 | 1.07 | 142.46 | 137.45 | 3.31 | 20295.82 | 18892.31 | 10.98 | 7.92 | 1.72 | 1.04 | 62.79 | 2.95 | 1.07 | 1.01 | 11.19 | 1.18 | 1.02 | 125.29 | 1.40 | -0.03 | 5.96 | 1.04 | 0.00 | 35.55 | 1.09 |
| 25 | 28.58 | -9.69 | 11.13 | 0.72 | 93.80 | 123.88 | 0.52 | 1.02 | 1.03 | 1.04 | 1.04 | 1.06 | 1.08 | 141.89 | 136.94 | 3.30 | 20132.26 | 18752.29 | 10.88 | 8.12 | 1.42 | 0.99 | 65.96 | 2.01 | 0.99 | 1.01 | 11.25 | 1.21 | 1.03 | 126.55 | 1.46 | -0.02 | 5.94 | 1.06 | 0.00 | 35.29 | 1.13 |
| 25 | 28.58 | -25.82 | -2.95 | 0.71 | 666.52 | 8.68 | 0.50 | 0.99 | 1.00 | 0.99 | 0.97 | 1.00 | 0.98 | 144.43 | 138.74 | 3.41 | 20860.80 | 19248.18 | 11.63 | 7.86 | 1.81 | 1.04 | 61.72 | 3.26 | 1.08 | 0.97 | 11.60 | 1.19 | 0.93 | 134.59 | 1.42 | 0.01 | 6.08 | 1.10 | 6.40E-005 | 36.93 | 1.22 |
| 25 | 28.58 | -14.64 | 7.29 | 0.72 | 214.45 | 53.17 | 0.52 | 1.02 | 1.03 | 1.04 | 1.04 | 1.07 | 1.08 | 141.39 | 135.89 | 3.27 | 19991.67 | 18465.66 | 10.66 | 7.98 | 1.63 | 1.02 | 63.70 | 2.66 | 1.04 | 1.03 | 11.17 | 1.19 | 1.05 | 124.80 | 1.41 | -0.03 | 5.94 | 1.05 | 0.00 | 35.32 | 1.11 |
| 25 | 24.27 | -10.11 | 8.62 | 0.72 | 102.27 | 74.27 | 0.52 | 1.02 | 1.03 | 1.04 | 1.04 | 1.06 | 1.08 | 138.63 | 134.05 | 3.15 | 19218.69 | 17969.08 | 9.95 | 8.12 | 1.42 | 1.00 | 65.93 | 2.01 | 1.01 | 1.01 | 11.12 | 1.16 | 1.02 | 123.71 | 1.33 | -0.01 | 5.98 | 1.06 | 0.00 | 35.79 | 1.12 |
| 25 | 52.85 | -8.11 | 10.02 | 0.72 | 65.76 | 100.46 | 0.52 | 1.00 | 1.01 | 1.01 | 1.00 | 1.02 | 1.03 | 138.11 | 133.47 | 3.14 | 19075.73 | 17814.16 | 9.83 | 7.99 | 1.53 | 1.00 | 63.88 | 2.34 | 1.00 | 0.98 | 11.43 | 1.10 | 0.95 | 130.62 | 1.20 | 0.01 | 6.04 | 1.06 | 3.60E-005 | 36.47 | 1.12 |
| 25 | 52.85 | -12.91 | 7.83 | 0.72 | 166.75 | 61.25 | 0.52 | 1.03 | 1.04 | 1.05 | 1.06 | 1.08 | 1.11 | 138.53 | 134.52 | 3.16 | 19191.12 | 18096.73 | 9.97 | 8.01 | 1.57 | 1.03 | 64.18 | 2.47 | 1.05 | 1.01 | 11.02 | 1.14 | 1.03 | 121.39 | 1.29 | -1.39E-017 | 6.06 | 1.07 | 1.93E-034 | 36.77 | 1.14 |
| 25 | 52.85 | -10.64 | 9.26 | 0.72 | 113.17 | 85.67 | 0.52 | 1.01 | 1.02 | 1.02 | 1.01 | 1.03 | 1.04 | 140.57 | 135.74 | 3.22 | 19760.88 | 18423.99 | 10.39 | 7.94 | 1.67 | 1.02 | 63.07 | 2.78 | 1.04 | 0.97 | 11.67 | 1.12 | 0.94 | 136.24 | 1.26 | 0.00 | 6.05 | 1.06 | 4.00E-006 | 36.58 | 1.12 |
| 25 | 52.85 | -6.03 | 14.37 | 0.73 | 36.39 | 206.50 | 0.53 | 1.01 | 1.02 | 1.03 | 1.03 | 1.04 | 1.06 | 140.22 | 135.50 | 3.22 | 19660.75 | 18360.71 | 10.36 | 7.95 | 1.62 | 1.01 | 63.17 | 2.62 | 1.02 | 0.98 | 11.33 | 1.15 | 0.97 | 128.27 | 1.33 | -0.02 | 5.94 | 1.05 | 0.00 | 35.24 | 1.09 |
| 25 | 48.54 | -3.21 | 16.31 | 0.73 | 10.30 | 265.95 | 0.53 | 0.99 | 1.00 | 1.00 | 0.99 | 1.01 | 1.01 | 135.68 | 130.95 | 3.02 | 18410.12 | 17148.37 | 9.15 | 8.03 | 1.52 | 0.99 | 64.49 | 2.32 | 0.97 | 1.06 | 10.79 | 1.21 | 1.13 | 116.47 | 1.47 | -0.01 | 5.98 | 1.04 | 0.00 | 35.79 | 1.07 |
| 25 | 6.98 | 0.15 | 19.61 | 0.73 | 0.02 | 384.36 | 0.54 | 1.02 | 1.03 | 1.04 | 1.05 | 1.05 | 1.09 | 139.44 | 135.03 | 3.19 | 19443.65 | 18232.10 | 10.18 | 8.01 | 1.55 | 1.00 | 64.11 | 2.40 | 1.01 | 0.97 | 11.31 | 1.14 | 0.94 | 128.02 | 1.29 | -0.04 | 5.90 | 1.03 | 0.00 | 34.77 | 1.07 |
| 25 | 0 | -20.70 | 2.99 | 0.72 | 428.53 | 8.91 | 0.51 | 1.04 | 1.05 | 1.06 | 1.08 | 1.10 | 1.13 | 140.37 | 136.18 | 3.23 | 19704.66 | 18544.39 | 10.46 | 7.91 | 1.80 | 1.07 | 62.57 | 3.23 | 1.14 | 1.01 | 10.96 | 1.16 | 1.02 | 120.06 | 1.35 | -0.01 | 6.07 | 1.07 | 0.00 | 36.80 | 1.14 |
| 25 | 20.78 | -12.19 | 12.68 | 0.73 | 148.67 | 160.81 | 0.53 | 1.02 | 1.03 | 1.04 | 1.04 | 1.05 | 1.07 | 146.57 | 140.73 | 3.48 | 21483.56 | 19804.43 | 12.13 | 8.03 | 1.57 | 1.01 | 64.50 | 2.48 | 1.03 | 0.98 | 11.42 | 1.18 | 0.97 | 130.52 | 1.39 | -0.03 | 5.99 | 1.06 | 0.00 | 35.82 | 1.12 |
| 25 | 4.31 | -5.24 | 16.95 | 0.73 | 27.43 | 287.30 | 0.53 | 1.02 | 1.03 | 1.04 | 1.04 | 1.06 | 1.07 | 140.23 | 135.40 | 3.23 | 19665.01 | 18333.08 | 10.45 | 8.05 | 1.52 | 1.00 | 64.83 | 2.31 | 0.99 | 1.01 | 11.25 | 1.18 | 1.01 | 126.55 | 1.40 | -0.04 | 5.91 | 1.04 | 0.00 | 34.91 | 1.08 |
| 50 | 0.84 | 10.44 | 33.56 | 0.75 | 109.01 | 1126.01 | 0.56 | 0.98 | 0.99 | 0.99 | 0.97 | 0.97 | 0.99 | 143.07 | 137.67 | 3.34 | 20467.79 | 18951.84 | 11.18 | 8.02 | 1.56 | 0.96 | 64.28 | 2.44 | 0.93 | 0.99 | 11.68 | 1.21 | 0.99 | 136.45 | 1.47 | 0.00 | 5.96 | 1.05 | 1.00E-006 | 35.52 | 1.09 |
| 50 | 49.38 | -15.80 | 7.82 | 0.72 | 249.55 | 61.20 | 0.52 | 1.02 | 1.03 | 1.04 | 1.03 | 1.06 | 1.07 | 142.46 | 137.45 | 3.31 | 20295.82 | 18892.31 | 10.98 | 7.92 | 1.72 | 1.04 | 62.79 | 2.95 | 1.07 | 1.01 | 11.19 | 1.18 | 1.02 | 125.29 | 1.40 | -0.03 | 5.96 | 1.04 | 0.00 | 35.55 | 1.09 |
| 50 | 28.60 | -9.69 | 11.13 | 0.72 | 93.80 | 123.88 | 0.52 | 1.02 | 1.03 | 1.04 | 1.04 | 1.06 | 1.08 | 141.89 | 136.94 | 3.30 | 20132.26 | 18752.29 | 10.88 | 8.12 | 1.42 | 0.99 | 65.96 | 2.01 | 0.99 | 1.01 | 11.25 | 1.21 | 1.03 | 126.55 | 1.46 | -0.02 | 5.94 | 1.06 | 0.00 | 35.29 | 1.13 |
| 50 | 28.60 | -25.82 | -2.95 | 0.71 | 666.52 | 8.68 | 0.50 | 0.99 | 1.00 | 0.99 | 0.97 | 1.00 | 0.98 | 144.43 | 138.74 | 3.41 | 20860.80 | 19248.18 | 11.63 | 7.86 | 1.81 | 1.04 | 61.72 | 3.26 | 1.08 | 0.97 | 11.60 | 1.19 | 0.93 | 134.59 | 1.42 | 0.01 | 6.08 | 1.10 | 6.40E-005 | 36.93 | 1.22 |
| 50 | 28.60 | -14.64 | 7.29 | 0.72 | 214.45 | 53.17 | 0.52 | 1.02 | 1.03 | 1.04 | 1.04 | 1.07 | 1.08 | 141.39 | 135.89 | 3.27 | 19991.67 | 18465.66 | 10.66 | 7.98 | 1.63 | 1.02 | 63.70 | 2.66 | 1.04 | 1.03 | 11.17 | 1.19 | 1.05 | 124.80 | 1.41 | -0.03 | 5.94 | 1.05 | 0.00 | 35.32 | 1.11 |
| 50 | 24.28 | -10.11 | 8.62 | 0.72 | 102.27 | 74.27 | 0.52 | 1.02 | 1.03 | 1.04 | 1.04 | 1.06 | 1.08 | 138.63 | 134.05 | 3.15 | 19218.69 | 17969.08 | 9.95 | 8.12 | 1.42 | 1.00 | 65.93 | 2.01 | 1.01 | 1.01 | 11.12 | 1.16 | 1.02 | 123.71 | 1.33 | -0.01 | 5.98 | 1.06 | 0.00 | 35.79 | 1.12 |
| 50 | 52.88 | -8.11 | 10.02 | 0.72 | 65.76 | 100.46 | 0.52 | 1.00 | 1.01 | 1.01 | 1.00 | 1.02 | 1.03 | 138.11 | 133.47 | 3.14 | 19075.73 | 17814.16 | 9.83 | 7.99 | 1.53 | 1.00 | 63.88 | 2.34 | 1.00 | 0.98 | 11.43 | 1.10 | 0.95 | 130.62 | 1.20 | 0.01 | 6.04 | 1.06 | 3.60E-005 | 36.47 | 1.12 |
| 50 | 52.88 | -12.91 | 7.83 | 0.72 | 166.75 | 61.25 | 0.52 | 1.03 | 1.04 | 1.05 | 1.06 | 1.08 | 1.11 | 138.53 | 134.52 | 3.16 | 19191.12 | 18096.73 | 9.97 | 8.01 | 1.57 | 1.03 | 64.18 | 2.47 | 1.05 | 1.01 | 11.02 | 1.14 | 1.03 | 121.39 | 1.29 | -1.39E-017 | 6.06 | 1.07 | 1.93E-034 | 36.77 | 1.14 |
| 50 | 52.88 | -10.64 | 9.26 | 0.72 | 113.17 | 85.67 | 0.52 | 1.01 | 1.02 | 1.02 | 1.01 | 1.03 | 1.04 | 140.57 | 135.74 | 3.22 | 19760.88 | 18423.99 | 10.39 | 7.94 | 1.67 | 1.02 | 63.07 | 2.78 | 1.04 | 0.97 | 11.67 | 1.12 | 0.94 | 136.24 | 1.26 | 0.00 | 6.05 | 1.06 | 4.00E-006 | 36.58 | 1.12 |
| 50 | 52.88 | -6.03 | 14.37 | 0.73 | 36.39 | 206.50 | 0.53 | 1.01 | 1.02 | 1.03 | 1.03 | 1.04 | 1.06 | 140.22 | 135.50 | 3.22 | 19660.75 | 18360.71 | 10.36 | 7.95 | 1.62 | 1.01 | 63.17 | 2.62 | 1.02 | 0.98 | 11.33 | 1.15 | 0.97 | 128.27 | 1.33 | -0.02 | 5.94 | 1.05 | 0.00 | 35.24 | 1.09 |
| 50 | 48.56 | -3.21 | 16.31 | 0.73 | 10.30 | 265.95 | 0.53 | 0.99 | 1.00 | 1.00 | 0.99 | 1.01 | 1.01 | 135.68 | 130.95 | 3.02 | 18410.12 | 17148.37 | 9.15 | 8.03 | 1.52 | 0.99 | 64.49 | 2.32 | 0.97 | 1.06 | 10.79 | 1.21 | 1.13 | 116.47 | 1.47 | -0.01 | 5.98 | 1.04 | 0.00 | 35.79 | 1.07 |
| 50 | 6.98 | 0.15 | 19.61 | 0.73 | 0.02 | 384.36 | 0.54 | 1.02 | 1.03 | 1.04 | 1.05 | 1.05 | 1.09 | 139.44 | 135.03 | 3.19 | 19443.65 | 18232.10 | 10.18 | 8.01 | 1.55 | 1.00 | 64.11 | 2.40 | 1.01 | 0.97 | 11.31 | 1.14 | 0.94 | 128.02 | 1.29 | -0.04 | 5.90 | 1.03 | 0.00 | 34.77 | 1.07 |
| 50 | 0 | -20.70 | 2.99 | 0.72 | 428.53 | 8.91 | 0.51 | 1.04 | 1.05 | 1.06 | 1.08 | 1.10 | 1.13 | 140.37 | 136.18 | 3.23 | 19704.66 | 18544.39 | 10.46 | 7.91 | 1.80 | 1.07 | 62.57 | 3.23 | 1.14 | 1.01 | 10.96 | 1.16 | 1.02 | 120.06 | 1.35 | -0.01 | 6.07 | 1.07 | 0.00 | 36.80 | 1.14 |
| 50 | 20.79 | -12.19 | 12.68 | 0.73 | 148.67 | 160.81 | 0.53 | 1.02 | 1.03 | 1.04 | 1.04 | 1.05 | 1.07 | 146.57 | 140.73 | 3.48 | 21483.56 | 19804.43 | 12.13 | 8.03 | 1.57 | 1.01 | 64.50 | 2.48 | 1.03 | 0.98 | 11.42 | 1.18 | 0.97 | 130.52 | 1.39 | -0.03 | 5.99 | 1.06 | 0.00 | 35.82 | 1.12 |
| 50 | 4.32 | -5.24 | 16.95 | 0.73 | 27.43 | 287.30 | 0.53 | 1.02 | 1.03 | 1.04 | 1.04 | 1.06 | 1.07 | 140.23 | 135.40 | 3.23 | 19665.01 | 18333.08 | 10.45 | 8.05 | 1.52 | 1.00 | 64.83 | 2.31 | 0.99 | 1.01 | 11.25 | 1.18 | 1.01 | 126.55 | 1.40 | -0.04 | 5.91 | 1.04 | 0.00 | 34.91 | 1.08 |
| 100 | 0.84 | 10.44 | 33.56 | 0.75 | 109.01 | 1126.01 | 0.56 | 0.98 | 0.99 | 0.99 | 0.97 | 0.97 | 0.99 | 143.07 | 137.67 | 3.34 | 20467.79 | 18951.84 | 11.18 | 8.02 | 1.56 | 0.96 | 64.28 | 2.44 | 0.93 | 0.99 | 11.68 | 1.21 | 0.99 | 136.45 | 1.47 | 0.00 | 5.96 | 1.05 | 1.00E-006 | 35.52 | 1.09 |
| 100 | 49.39 | -15.80 | 7.82 | 0.72 | 249.55 | 61.20 | 0.52 | 1.02 | 1.03 | 1.04 | 1.03 | 1.06 | 1.07 | 142.46 | 137.45 | 3.31 | 20295.82 | 18892.31 | 10.98 | 7.92 | 1.72 | 1.04 | 62.79 | 2.95 | 1.07 | 1.01 | 11.19 | 1.18 | 1.02 | 125.29 | 1.40 | -0.03 | 5.96 | 1.04 | 0.00 | 35.55 | 1.09 |
| 100 | 28.60 | -9.69 | 11.13 | 0.72 | 93.80 | 123.88 | 0.52 | 1.02 | 1.03 | 1.04 | 1.04 | 1.06 | 1.08 | 141.89 | 136.94 | 3.30 | 20132.26 | 18752.29 | 10.88 | 8.12 | 1.42 | 0.99 | 65.96 | 2.01 | 0.99 | 1.01 | 11.25 | 1.21 | 1.03 | 126.55 | 1.46 | -0.02 | 5.94 | 1.06 | 0.00 | 35.29 | 1.13 |
| 100 | 28.60 | -25.82 | -2.95 | 0.71 | 666.52 | 8.68 | 0.50 | 0.99 | 1.00 | 0.99 | 0.97 | 1.00 | 0.98 | 144.43 | 138.74 | 3.41 | 20860.80 | 19248.18 | 11.63 | 7.86 | 1.81 | 1.04 | 61.72 | 3.26 | 1.08 | 0.97 | 11.60 | 1.19 | 0.93 | 134.59 | 1.42 | 0.01 | 6.08 | 1.10 | 6.40E-005 | 36.93 | 1.22 |
| 100 | 28.60 | -14.64 | 7.29 | 0.72 | 214.45 | 53.17 | 0.52 | 1.02 | 1.03 | 1.04 | 1.04 | 1.07 | 1.08 | 141.39 | 135.89 | 3.27 | 19991.67 | 18465.66 | 10.66 | 7.98 | 1.63 | 1.02 | 63.70 | 2.66 | 1.04 | 1.03 | 11.17 | 1.19 | 1.05 | 124.80 | 1.41 | -0.03 | 5.94 | 1.05 | 0.00 | 35.32 | 1.11 |
| 100 | 24.28 | -10.11 | 8.62 | 0.72 | 102.27 | 74.27 | 0.52 | 1.02 | 1.03 | 1.04 | 1.04 | 1.06 | 1.08 | 138.63 | 134.05 | 3.15 | 19218.69 | 17969.08 | 9.95 | 8.12 | 1.42 | 1.00 | 65.93 | 2.01 | 1.01 | 1.01 | 11.12 | 1.16 | 1.02 | 123.71 | 1.33 | -0.01 | 5.98 | 1.06 | 0.00 | 35.79 | 1.12 |
| 100 | 52.88 | -8.11 | 10.02 | 0.72 | 65.76 | 100.46 | 0.52 | 1.00 | 1.01 | 1.01 | 1.00 | 1.02 | 1.03 | 138.11 | 133.47 | 3.14 | 19075.73 | 17814.16 | 9.83 | 7.99 | 1.53 | 1.00 | 63.88 | 2.34 | 1.00 | 0.98 | 11.43 | 1.10 | 0.95 | 130.62 | 1.20 | 0.01 | 6.04 | 1.06 | 3.60E-005 | 36.47 | 1.12 |

| External glucose, mM | metabolic flux, mmol min$^{-1}$ | $P_{ave}$ WV1-1 | $P_{ave}$ WV1-2 | $P_{ave}$ WV1-3 | ($P_{ave}$ WV1-1)^2 | ($P_{ave}$ WV1-2)^2 | ($P_{ave}$ WV1-3)^2 | $P_{ave}$ WV2-1 | $P_{ave}$ WV2-2 | $P_{ave}$ WV2-3 | ($P_{ave}$ WV2-1)^2 | ($P_{ave}$ WV2-2)^2 | ($P_{ave}$ WV2-3)^2 | $P_{ave}$ WV3-1 | $P_{ave}$ WV3-2 | $P_{ave}$ WV3-3 | ($P_{ave}$ WV3-1)^2 | ($P_{ave}$ WV3-2)^2 | ($P_{ave}$ WV3-3)^2 | $P_{ave}$ WV5-1 | $P_{ave}$ WV5-2 | $P_{ave}$ WV5-3 | ($P_{ave}$ WV5-1)^2 | ($P_{ave}$ WV5-2)^2 | ($P_{ave}$ WV5-3)^2 | $P_{ave}$ WV6-1 | $P_{ave}$ WV6-2 | $P_{ave}$ WV6-3 | ($P_{ave}$ WV6-1)^2 | ($P_{ave}$ WV6-2)^2 | ($P_{ave}$ WV6-3)^2 | $P_{ave}$ WV7-1 | $P_{ave}$ WV 7-2 | $P_{ave}$ WV 7-3 | ($P_{ave}$ WV7-1)^2 | ($P_{ave}$ WV7-2)^2 | ($P_{ave}$ WV7-3)^2 |
|---|---|---|---|---|---|---|---|---|---|---|---|---|---|---|---|---|---|---|---|---|---|---|---|---|---|---|---|---|---|---|---|---|---|---|---|---|---|
| 100 | 52.88 | -12.91 | 7.83 | 0.72 | 166.75 | 61.25 | 0.52 | 1.03 | 1.04 | 1.05 | 1.06 | 1.08 | 1.11 | 138.53 | 134.52 | 3.16 | 19191.12 | 18096.73 | 9.97 | 8.01 | 1.57 | 1.03 | 64.18 | 2.47 | 1.05 | 1.01 | 11.02 | 1.14 | 1.03 | 121.39 | 1.29 | -1.39E-017 | 6.06 | 1.07 | 1.93E-034 | 36.77 | 1.14 |
| 100 | 52.88 | -10.64 | 9.26 | 0.72 | 113.17 | 85.67 | 0.52 | 1.01 | 1.02 | 1.02 | 1.01 | 1.03 | 1.04 | 140.57 | 135.74 | 3.22 | 19760.88 | 18423.99 | 10.39 | 7.94 | 1.67 | 1.02 | 63.07 | 2.78 | 1.04 | 0.97 | 11.67 | 1.12 | 0.94 | 136.24 | 1.26 | 0.00 | 6.05 | 1.06 | 4.00E-006 | 36.58 | 1.12 |
| 100 | 52.88 | -6.03 | 14.37 | 0.73 | 36.39 | 206.50 | 0.53 | 1.01 | 1.02 | 1.03 | 1.03 | 1.04 | 1.06 | 140.22 | 135.50 | 3.22 | 19660.75 | 18360.71 | 10.36 | 7.95 | 1.62 | 1.01 | 63.17 | 2.62 | 1.02 | 0.98 | 11.33 | 1.15 | 0.97 | 128.27 | 1.33 | -0.02 | 5.94 | 1.05 | 0.00 | 35.24 | 1.09 |
| 100 | 48.56 | -3.21 | 16.31 | 0.73 | 10.30 | 265.95 | 0.53 | 0.99 | 1.00 | 1.00 | 0.99 | 1.01 | 1.01 | 135.68 | 130.95 | 3.02 | 18410.12 | 17148.37 | 9.15 | 8.03 | 1.52 | 0.99 | 64.49 | 2.32 | 0.97 | 1.06 | 10.79 | 1.21 | 1.13 | 116.47 | 1.47 | -0.01 | 5.98 | 1.04 | 0.00 | 35.79 | 1.07 |
| 100 | 6.98 | 0.15 | 19.61 | 0.73 | 0.02 | 384.36 | 0.54 | 1.02 | 1.03 | 1.04 | 1.05 | 1.05 | 1.09 | 139.44 | 135.03 | 3.19 | 19443.65 | 18232.10 | 10.18 | 8.01 | 1.55 | 1.00 | 64.11 | 2.40 | 1.01 | 0.97 | 11.31 | 1.14 | 0.94 | 128.02 | 1.29 | -0.04 | 5.90 | 1.03 | 0.00 | 34.77 | 1.07 |
| 100 | -5.68E-014 | -20.70 | 2.99 | 0.72 | 428.53 | 8.91 | 0.51 | 1.04 | 1.05 | 1.06 | 1.08 | 1.10 | 1.13 | 140.37 | 136.18 | 3.23 | 19704.66 | 18544.39 | 10.46 | 7.91 | 1.80 | 1.07 | 62.57 | 3.23 | 1.14 | 1.01 | 10.96 | 1.16 | 1.02 | 120.06 | 1.35 | -0.01 | 6.07 | 1.07 | 0.00 | 36.80 | 1.14 |
| 100 | 20.79 | -12.19 | 12.68 | 0.73 | 148.67 | 160.81 | 0.53 | 1.02 | 1.03 | 1.04 | 1.04 | 1.05 | 1.07 | 146.57 | 140.73 | 3.48 | 21483.56 | 19804.43 | 12.13 | 8.03 | 1.57 | 1.01 | 64.50 | 2.48 | 1.03 | 0.98 | 11.42 | 1.18 | 0.97 | 130.52 | 1.39 | -0.03 | 5.99 | 1.06 | 0.00 | 35.82 | 1.12 |
| 100 | 4.32 | -5.24 | 16.95 | 0.73 | 27.43 | 287.30 | 0.53 | 1.02 | 1.03 | 1.04 | 1.04 | 1.06 | 1.07 | 140.23 | 135.40 | 3.23 | 19665.01 | 18333.08 | 10.45 | 8.05 | 1.52 | 1.00 | 64.83 | 2.31 | 0.99 | 1.01 | 11.25 | 1.18 | 1.01 | 126.55 | 1.40 | -0.04 | 5.91 | 1.04 | 0.00 | 34.91 | 1.08 |

[a] estimated by the Hynne's kinetic model as described in Material and Methods;

[b] in accordance with 10 VARIMAX-derived scales (Georgiev, 2009 [18]) which represent generalized numerical scores for physical-chemical and structural features of amino acids.
