# Supplementary material for: Relationship between Metabolic Fluxes and Sequence-Derived Properties of Enzymes
Source: Int Sch Res Notices. 2014 Oct 29;2014:817102. doi: 10.1155/2014/817102 (PMC4897147; doi:10.1155/2014/817102)
Supplement: Supplementary file 1 — Supplementary information 1: Table S1-1: The dataset used in multivariate analysis which comprises metabolic fluxes and numerical vectors representing the average amino acid (AA) property for each enzyme sequence (Teusink's kinetic model, VARIMAX scales). Table S1-2: The dataset used in multivariate analysis which comprises metabolic fluxes and numerical vectors representing the average AA property for each enzyme sequence (Hynne's kinetic model, VARIMAX scales). Table S1-3: The dataset used in multivariate analysis which comprises metabolic fluxes and numerical vectors representing the average AA property for each enzyme sequence (Teusink's kinetic model, specific AA indices). Table S1-4: The dataset used in multivariate analysis which comprises metabolic fluxes and numerical vectors representing the average AA property for each enzyme sequence (Hynne's kinetic model, specific AA indices). Supplementary information 2: Figure S2: The linear plots for the metabolic fluxes of the yeast Saccharomyces cerevisiae glycolysis pathway estimated by kinetic models against those predicted by linear regression models I-IV and by the leave-one-out cross-validation (LOOCV) of the models. Table S2-1: The variance analysis of the regression models. Table S2-2: Standard errors and confidence intervals for the linear regression models. Supplementary information 3: Table S3: Elements and the statistical indices for multiple linear regression model which links the values of metabolic fluxes and the average AA properties of the yeast Saccharomyces cerevisiae enzyme sequences expressed according to the specific AA indices. Figure S3: The linear plot for the metabolic fluxes of the yeast Saccharomyces cerevisiae glycolysis pathway estimated by kinetic models against those predicted by linear regression model. [file 817102.f1.zip › manuscript_SI2.pdf]

RELATIONSHIP BETWEEN METABOLIC FLUXES AND SEQUENCE-DERIVED PROPERTIES OF ENZYMES

Peteris Zikmanis, Inara Kampenusa
Institute of Microbiology and Biotechnology, University of Latvia, Kronvalda Boulevard 4, LV-1010, Riga, Latvia; Correspondence should be addressed to Peteris Zikmanis; peteris.zikmanis@lu.lv

Supplementary information 2

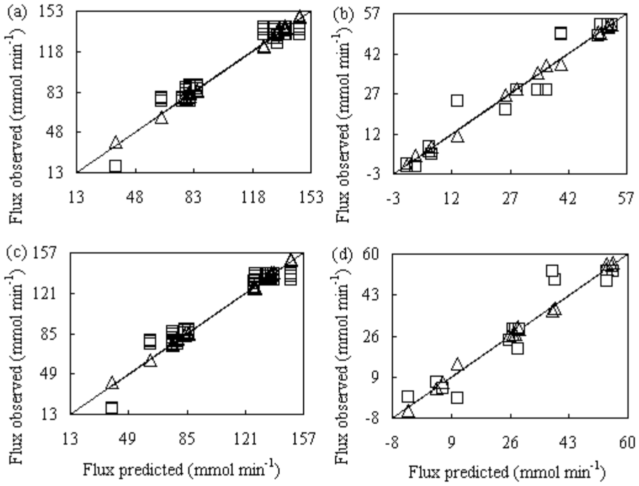

Fig. S2 The linear plots (a, b, c, and d) for the metabolic fluxes of the yeast *Saccharomyces cerevisiae* glycolysis pathway estimated by kinetic models against those predicted by linear regression models I-IV, respectively, (□) and by the leave-one-out cross-validation (LOOCV) of the models (Δ) (Table 2, Table 3) with the $R^2_{adj.}$ values of 91.69, 91.46, 91.95 and 85.61 percent, respectively.

Table S2-1. The variance analysis of the regression models. Multiple linear regression models linking the values of metabolic fluxes and the average AA properties of the yeast *Saccharomyces cerevisiae* enzyme sequences.

Model	Variables: dependent	Variables: independent[a]	Variance source	Sum of squares	Df	Mean square	F-ratio	*p*-value
I	metabolic flux	P_{ave}VW7, P_{ave}VW5, $(P_{ave}\text{VW1})^{2}$,	model	48821.70	7	6974.53	82.81	0.0000
	(Teusink's [a] model)	$(P_{ave}\text{VW5})^{2}$, $(P_{ave}\text{VW2})^{2}$, P_{ave}VW6,	*residual*	2863.50	34	84.22		
		P_{ave}VW1	total	51685.20	41			
II	metabolic flux	$(P_{ave}\text{VW2})^{2}$, P_{ave}VW7, P_{ave}VW1,	model	16603.00	8	2075.37	73.09	0.0000
	(Hynne's model)	$(P_{ave}\text{VW1})^{2}$, $(P_{ave}\text{VW5})^{2}$, P_{ave}VW5,	*residual*	1022.26	36	28.40		
		P_{ave}VW6, $(P_{ave}\text{VW3})^{2}$	total	17625.20	44			
III	metabolic flux	$(P_{ave}\text{VW7-1})^{2}$, P_{ave}VW2-3, (P_{ave}VW5-	model	48594.00	5	9718.80	113.18	0.0000
	(Teusink's model)	1)^{2}, $(P_{ave}\text{VW1-1})^{2}$, P_{ave}VW3-1	*residual*	3091.21	36	85.87		
			total	51685.20	41			
IV	metabolic flux	$(P_{ave}\text{VW1-1})^{2}$, P_{ave}VW1-3, P_{ave}VW2-	model	15907.30	7	2272.47	48.94	0.0000
	(Hynne's model)	1, $(P_{ave}\text{VW5-1})^{2}$, $(P_{ave}\text{VW7-1})^{2}$,	*residual*	1717.95	37	46.43		
		P_{ave}VW6-1, P_{ave}VW3-1	total	17625.20	44	17625.20		

[a] as specified in the Table 1

Table S2-2. Standard errors and confidence intervals for the linear regression models.

Model	±S.E.[b], mmol min $^{-1}$	±CI[b], mmol min $^{-1}$ 95%	99%
I	9.177	18.650	25.039
II	5.329	10.808	14.492
III	9.266	18.792	25.199
IV	6.814	13.806	18.503

[a] the standard error of the estimate for the regression model
[b] the confidence interval for predicted values of a linear regression model
