# Supplementary material for: Relationship between Metabolic Fluxes and Sequence-Derived Properties of Enzymes
Source: Int Sch Res Notices. 2014 Oct 29;2014:817102. doi: 10.1155/2014/817102 (PMC4897147; doi:10.1155/2014/817102)
Supplement: Supplementary file 1 — Supplementary information 1: Table S1-1: The dataset used in multivariate analysis which comprises metabolic fluxes and numerical vectors representing the average amino acid (AA) property for each enzyme sequence (Teusink's kinetic model, VARIMAX scales). Table S1-2: The dataset used in multivariate analysis which comprises metabolic fluxes and numerical vectors representing the average AA property for each enzyme sequence (Hynne's kinetic model, VARIMAX scales). Table S1-3: The dataset used in multivariate analysis which comprises metabolic fluxes and numerical vectors representing the average AA property for each enzyme sequence (Teusink's kinetic model, specific AA indices). Table S1-4: The dataset used in multivariate analysis which comprises metabolic fluxes and numerical vectors representing the average AA property for each enzyme sequence (Hynne's kinetic model, specific AA indices). Supplementary information 2: Figure S2: The linear plots for the metabolic fluxes of the yeast Saccharomyces cerevisiae glycolysis pathway estimated by kinetic models against those predicted by linear regression models I-IV and by the leave-one-out cross-validation (LOOCV) of the models. Table S2-1: The variance analysis of the regression models. Table S2-2: Standard errors and confidence intervals for the linear regression models. Supplementary information 3: Table S3: Elements and the statistical indices for multiple linear regression model which links the values of metabolic fluxes and the average AA properties of the yeast Saccharomyces cerevisiae enzyme sequences expressed according to the specific AA indices. Figure S3: The linear plot for the metabolic fluxes of the yeast Saccharomyces cerevisiae glycolysis pathway estimated by kinetic models against those predicted by linear regression model. [file 817102.f1.zip › manuscript_SI3.pdf]

RELATIONSHIP BETWEEN METABOLIC FLUXES AND SEQUENCE-DERIVED PROPERTIES OF ENZYMES

Peteris Zikmanis, Inara Kampenusa
Institute of Microbiology and Biotechnology, University of Latvia, Kronvalda Boulevard 4, LV-1010, Riga, Latvia; Correspondence should be addressed to Peteris Zikmanis; peteris.zikmanis@lu.lv

Supplementary information 3

Table S3. Elements and the statistical indices for multiple linear regression model which links the values of metabolic fluxes and the average AA properties of the yeast *Saccharomyces cerevisiae* enzyme sequences expressed according to the AAindex scales.

Dependent [a] variable	Parameters [b]	Regression coefficient (standardized value)	S.E.	t value	P value	R^2 ,% $R^2_{adj.}$, %	VIF [c]
	constant	*43.4234*	*6.193*	*7.01*	0.0000	71.95 68.08	
metabolic flux	P_{ave}WV5-1	-3.2314 (-0.6118)	0.626	-5.16	0.0000		1.45
	P_{ave}WV8-1	8.0585 (0.3406)	2.842	2.84	0.0082		1.49
	P_{ave}VW10-1	-11.5014 (-1.0396)	1.828	-6.29	0.0000		2.82
	P_{ave}WV10-3	28.6417 (1.2986)	3.607	7.94	0.0000		2.76

[a] represent the values of metabolic fluxes for HXT, HXK, GPI, ALD, PFK, TIM, GAPDH, PGK, PGM, ENOL, PK, PDC, ADH, GPD, ATPase, AK and α,α- trehalose-phosphate synthase (Q00764) as specified in the "Material and Methods" and listed in Stanford *et al*., 2013 [25] for both the standard model built without regulatory information and the model with an integrated regulatory information.
[b] elements of multiple linear regression which represent the average AA property of the yeast *Saccharomyces cerevisiae* enzyme sequences and the constant (intercept) of equation, specified (Stanford *et al*., 2013 [25]) as follows:
P_{ave}WV5-1 (BUNA790101) represents α-NH chemical shifts (Bundi-Wuthrich, 1979);
P_{ave}WV8-1 (QIAN880117) represents weights for β-sheet at the window position of _3 (Qian-Sejnowski, 1988);
P_{ave}VW10-1 (MAXF760103) represents normalized frequency of zeta R (Maxfield-Scheraga, 1976);
P_{ave}WV10-3 (WERD780102) represents free energy change of epsilon(i) to epsilon(ex) (Wertz-Scheraga, 1978).
In brackets: the AA index accession numbers.
[c] the variance inflation factor which indicates the impact of collinearity between the independent variables (Kock and Lynn, 2012 [22]).

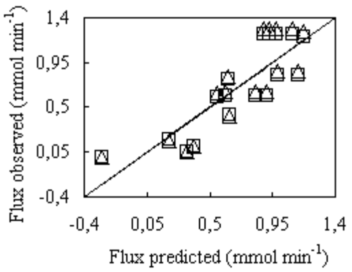

Fig. S3 The linear plot for the metabolic fluxes of the yeast *Saccharomyces cerevisiae* glycolysis pathway estimated by kinetic models (Stanford *et al*., 2013 [25]) against those predicted by linear regression model (Table S3). (□) – The standard model; (Δ) – The model with regulatory information. R=0.848, Kendall′s τ = 0.642 , Spearman′s ρ = 0.831.
